# Responsive Accumulation of Nanohybrids to Boost NIR‐Phototheranostics for Specific Tumor Imaging and Glutathione Depletion‐Enhanced Synergistic Therapy

**DOI:** 10.1002/advs.202205208

**Published:** 2022-11-14

**Authors:** Liangcan He, Nannan Zheng, Qinghui Wang, Jiarui Du, Shumin Wang, Zhiyue Cao, Zhantong Wang, Guanying Chen, Jing Mu, Shaoqin Liu, Xiaoyuan Chen

**Affiliations:** ^1^ School of Medicine and Health, Key Laboratory of Micro‐systems and Micro‐structures Manufacturing (Ministry of Education) Harbin Institute of Technology Harbin 150001 China; ^2^ Institute of Precision Medicine Peking University Shenzhen Hospital Shenzhen 518036 China; ^3^ School of Chemistry and Chemical Engineering Harbin Institute of Technology Harbin 150090 China; ^4^ Laboratory of Cellular Imaging and Macromolecular Biophysics National Institute of Biomedical Imaging and Bioengineering National Institutes of Health Bethesda MD 20892 USA; ^5^ Departments of Diagnostic Radiology Surgery Chemical and Biomolecular Engineering and Biomedical Engineering Yong Loo Lin School of Medicine and College of Design and Engineering National University of Singapore Singapore 117597 Singapore; ^6^ Institute of Molecular and Cell Biology Agency for Science, Technology, and Research (A*STAR) 61 Biopolis Drive, Proteos Singapore 138673 Singapore

**Keywords:** acidic‐responsive, glutathione depletion, metal‐organic frameworks, NIR‐II imaging, synergistic therapy

## Abstract

Dynamic regulation of nanoparticles in a controllable manner has great potential in various areas. Compared to the individual nanoparticles, the assembled nanoparticles exhibit superior properties and functions, which can be applied to achieve desirable performances. Here, a pH‐responsive i‐motif DNA‐mediated strategy to tailor the programmable behaviors of erbium‐based rare‐earth nanoparticles (ErNPs) decorated copper doped metal‐organic framework (CPM) nanohybrids (ECPM) under physiological conditions is reported. Within the acidic tumor microenvironment, the i‐motif DNA strands are able to form quadruplex structures, resulting in the assembly of nanohybrids and selective tumor accumulation, which further amplify the ErNPs downconversion emission (1550 nm) signal for imaging. Meanwhile, the ECPM matrix acts as a near‐infrared (NIR) photon‐activated reactive oxygen species (ROS) amplifier through the singlet oxygen generation of the matrix in combination with its ability of intracellular glutathione depletion upon irradiation. In short, this work displays a classical example of engineering of nanoparticles, which will manifest the importance of developing nanohybrids with structural programmability in biomedical applications.

## Graphical abstract

“Unity makes strength,” the smart use of MOF‐based nanohybrid (ECPM) for depleting the intracellular GSH and thus increasing the delivery efficiency of the ECPM is reported. The unique acidity‐responsive assembly of ECPM enables in situ clustering of the nanohybrid in the acidic tumor microenvironment (TME), which can achieve NIR‐activated tumor‐specific cancer theranostics with negligible side effects.

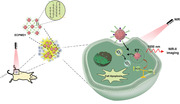

## 1 Introduction

The past decades have witnessed the explosive growth in the control of size, shape, composition, and physical and chemical properties of nanoparticles (NPs).^[^ ^1^ ^]^ More recently, ensembles of NPs with collective properties and distinct functions, compared to individual NPs, have lately raised high interest in this field. In particular, spatial regulation (i.e., tuning the distance) in a programmable manner provides the opportunity to explore their superior properties and functions, which holds great potential in the area of nanophotonics, nanoelectronics, and nanomedicine.^[^ ^2^ ^]^ Among the developed strategies, DNA nanotechnology stands out to organize nanomaterials in a controllable way. The rigid double helix could be formed according to the specific Watson–Crick base pairing rules, which has been widely used as a fascinating template for positioning of nanoparticles with nanoscale precision.^[^ ^2^, ^3^ ^]^ Intercalated motif (i‐motif) DNAs are four stranded quadruplex structure folded by the complementary cytosine rich sequences. By virtue of their pH‐sensitive feature, i‐motif DNAs can achieve the transformation from random coil to folded quadruplex structures under acidic pH conditions, which has been employed to construct pH sensors, pH‐responsive hydrogels and DNA motors, etc.^[^ ^4^ ^]^

In the field of nanomedicine, tailored nanostructures for efficient cellular uptake are critical for achieving various biological functions. It is generally accepted that relatively small particles within the size range of 5–100 nm easily penetrate into tumor area, while issues on the rapid clearance from the body also exist.^[^ ^5^ ^]^ In contrast, larger nanomaterials are able to reduce the clearance and prolong retention in the tumor, yet the inability of biodegradation limits their further applications.^[^ ^6^ ^]^ One promising proposed approach to improve the delivery efficiency is using the strategy of in situ self‐assembly. During the process of blood circulation, once smaller nanoparticles extravasate into the tumor region, nanoparticles surface changes in response to certain stimuli allow them to self‐assemble into larger particles.^[^ ^7^ ^]^ Such “size expansion” effect could easily trap nanoparticles within the local areas, hence increasing the tumor tissue uptake and minimizing the intravasation. To date, the responsive self‐assembly strategies for delivering imaging contrasts or therapeutic agents into the tumor site are still in their infancy, more efforts are needed to explore new ways of in situ assembly and how such assembled nanostructures can mediate their biological performances should be further investigated.^[^ ^8^ ^]^

Conceivably, glutathione (GSH) plays a very important role in maintaining various cellular processes including cellular metabolism and antioxidant defense. The highly expressed GSH (5–10 mm) in cancer cells might be one of the challenges for enhancing reactive oxygen species (ROS)‐mediated therapy efficacy.^[^ ^9^ ^]^ Moreover, the elevated level of GSH in cancer cells was revealed to increase resistance to photodynamic, chemo‐, and radiotherapies.^[^ ^10^ ^]^ Particularly, the intracellular GSH exhibits significant scavenging effect on reactive singlet oxygen produced by photo‐/sono‐sensitizers, increasing tumor resistance to oxidative stress and further diminishing the photo‐/sono‐dynamic efficacy. Therefore, it's highly desirable to develop strategy for circumventing the tumor resistance and thus improving the efficacy.

**Figure 1:**
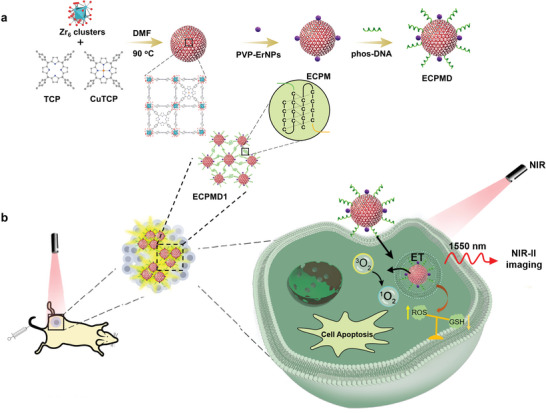
a) Schematic illustration of the synthesis of ECPM nanohybrid and its postsynthetic modification procedure. b) Scheme for the acidic tumor microenvironment responsive ECPM nanohybrids for augmenting their biological performances in NIR‐II emission region and efficacious cancer therapy achieved by GSH depletion‐enhanced phototherapy.

Here, we provided a DNA‐mediated responsive assembly strategy to achieve the increased localization of erbium‐based rare‐earth nanoparticles (ErNPs) decorated copper doped metal‐organic framework (CPM) nanohybrids (ECPM) at the tumor site for amplifying the imaging signal and boosting the therapeutic efficacy. The virus‐like ECPM was composed of inner CPM and outer ErNPs, and the surface was further modified with pH‐responsive i‐motif DNA strands via the metal–phosphate coordination interactions (**Figure** **1**). Such virus‐like ECPM NPs can be well dispersed in normal blood (pH ≈ 7.4). Upon exposure to the acidic tumor microenvironment, the DNA stands on ECPM surface tend to form the quadruplex structure via intermolecular interactions, thus mediating the self‐assembly of hybrid NPs into clusters. Under low energy photons excitation, the emitted visible photon from the ErNPs could be easily harvested by the porphyrin ligands in CPM for NIR‐induced photodynamic therapy. Yet, the integration of CPM had no effect on the down‐converted second near‐infrared II (NIR‐II) emission. In addition, CPM could deplete the level of intracellular GSH and amplify the oxidative stress for photodynamic therapy. Compared to dispersed ECPM system, the responsive ECPM assembly system could achieve selective tumor accumulation, enabling improved performances on the near‐infrared‐II (NIR‐II) imaging and therapeutic effect.

## 2 Results and Discussion

### 2.1 Synthesis and Characterization of ErNPs and CPM

**Figure 2:**
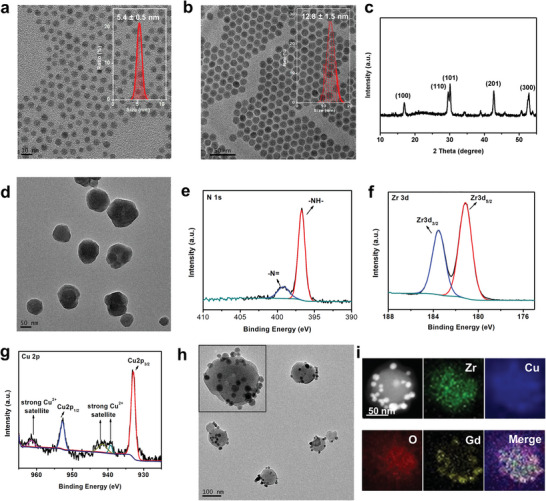
a) Energy level diagram of Er^3+^ and the excitation and corresponding emission pathways for simultaneous upconversion or downconversion emission. b) Transmission electron microscope (TEM) images of NaGd_0.9_F_4_, Er_0.1_ and c) the core–shell ErNPs (inset is the corresponding size statistics results). d) XRD patterns of ErNPs. e) TEM image of CPM. High‐resolution XPS spectra of f) N 1s, g) Zr 3d, and h) Cu 2p for CPM. i) TEM image of ECPM. High‐angle annular dark field scanning transmission electron microscope (STEM) image of a ECPM NP and its elemental mapping results (h).

Due to the rich energy level system of erbium (Er^3+^), in this work we used the Er^3+^ as the active dopant ion in our design, which could offer multiple excitation and emission pathways ranging from visible to NIR wavelengths. For this, the oleic acid capped core–shell NaGd_0.9_F_4_,Er_0.1_@NaGdF_4_ NPs (ErNPs) were firstly synthesized using a thermal decomposition method.^[^ ^11^ ^]^ As shown in **Figure** **2**a,b, the core–shell crystal structure was composed of NaGd_0.9_F_4_,Er_0.1_ core with a diameter around 5.4 nm and an inert layer of NaGdF_4_ shell, which could minimize surface and environmental quenching effect, thus improving visible light emission efficiency.^[^ ^12^ ^]^ The powder X‐ray diffraction (XRD) patterns confirmed the *β*‐phase structures (Figure 2c). The hydrophobic Er nanocrystals were then modified with polyvinylpyrrolidone (PVP) via a ligand exchange reaction to become hydrophilic for further use. The copper‐doped metal‐organic frameworks (denoted as CPM) were assembled by Cu(II) meso‐tetra (4‐carboxyphenyl) porphyrine/meso‐tetra (4‐carboxyphenyl) porphyrine (1:1) and zirconylchloride octahydrate (ZrOCl_2_•8H_2_O), which demonstrated spherical shape structure with a diameter of ≈80 nm (Figure 2d, and Figure S1, Supporting Information). The optical property of the CPM was measured by UV–vis absorption spectroscopy (Figure S2, Supporting Information). The Soret band around 420 nm was the characteristic peak of porphyrin ring in the mixture ligand. The CPM and meso‐tetra(4‐carboxyphenyl) porphine (TCP) gave four Q bands in the region of 510–660 nm, while the CuTCP ligand only showed two predominant Q bands in 540–590 nm.^[^ ^13^ ^]^ These results indicate that both the free TCP and CuTCP were well preserved in the CPM matrix.

To further explore the chemical structures of the CPM, the X‐ray photoelectron spectroscopy (XPS) measurement for CPM and high‐resolution analysis for Zr 3d, Cu 2p, and N 1s were carried out. As shown in Figure S3 (Supporting Information), the C1s signals were located at 284.6, 286.1, and 288.7 eV, which were ascribed to C = C, C–C/C–H, and C = N/C = O, respectively. The N 1s peaks around 396.8 and 399.2 eV were the typical features ascribed to the pyrrolic N and –N = in CuTCP ligand (Figure 2e). The signals of Zr 3d_5/2_ (181.2 eV), Zr 3d_3/2_ (183.6 eV), and Zr–O (528.7 eV) mainly corresponded to the Zr‐containing secondary building unit nodes (Figure 2f). The major peaks of Cu 2p_3/2_ at 932.9 and Cu 2p_1/2_at 952.7 eV in combination with the satellite peaks confirmed the existence of metallic copper in the pyrrolic groups (Figure 2g). In addition, the high‐resolution XPS spectrum of TPM (assembled by TCP and Zr_6_ clusters) in Figure S4 (Supporting Information) showed no shift in the binding energy for all the C 1s, N 1s, O 1s, and Zr 3d, indicating the TPM and CPM possessed the same coordination nodes. Moreover, the signals of –N = (399.2 eV) was much higher than that for CPM, indicating the absence of coordination of Cu^2+^ with the nitrogen in ligand.

Integration of photosensitizers with NIR‐light active NPs is one of the effective ways to overcome the limit of penetration depth in phototheranostics.^[^ ^12^ ^]^ While, the commonly used photosensitizer molecules encapsulation or loading strategies were largely held back by the low yield as well as leaching during the delivery process.^[^ ^14^ ^]^ The emergence of the structurally rigid porphyrin MOFs tends to address the above issues, as the chemical and physical properties of the active porphyrin ligands are well preserved in the MOFs matrix.^[^ ^15^ ^]^ Additionally, the advantages of MOFs matrix such as regular pores and channels are also beneficial for efficient diffusion of the reactant and products in confined microenvironment. Inspired by the outstanding performances of MOFs, the CPM was rationally integrated with luminescent ErNPs to form virus‐like ECPM nanohybrid structures, where the CPM acted as a NIR light photon harvester. For this, the ECPM nanohybrids were prepared by directly mixing CPM with PVP‐stabilized ErNPs at fixed ratio in *N*, *N*‐dimethylformamide (DMF) solution under stirring for overnight. The obtained ECPM nanohybrids demonstrated a virus‐like structure and there was almost no free ErNPs around (Figure 2h). As shown in Figure 2i, the high‐angle annular dark‐field scanning transmission electron microscopy (HAADF‐STEM) image clearly showed that Zr and O were homogenously distributed throughout the whole assembled ECPM, while the Gd from ErNPs was only distributed in the outer area, suggesting that the CPM core was surrounded by the outer ErNPs. In addition, the powder X‐ray diffraction (XRD) pattern confirmed that the ECPM nanohybrids were composed of both CPM and *β*‐phase ErNPs, and no peak assigned to other impurities was detected (Figure S5).

**Figure 3:**
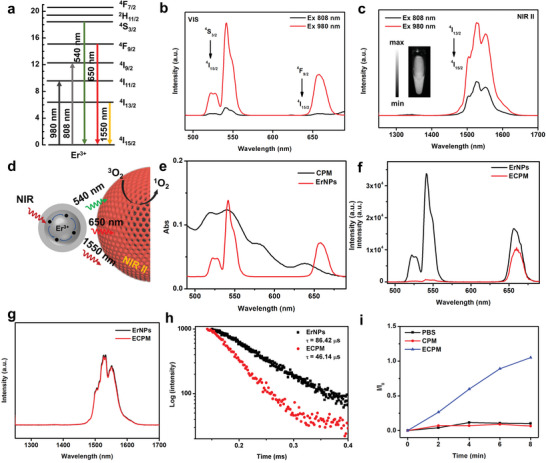
a) Energy level diagram of erbium and the excitation and corresponding emission pathways for simultaneous upconvertion or downconversion emission. b) UCL emission and c) DCL emission spectra of ErNPs under 808 and 980 nm excitation at similar powder density (the inset in (c) was the NIR‐II photo of ErNPs solution). d) Scheme of the energy transfer mechanism from ErNPs to CPM. e) UCL emission spectrum (red) of ErNPs and the UV–vis absorption spectrum (black) of the CPM. f) UCL emission and g) DCL emission spectra of ErNPs and ECPM. h) UCL decay curves of the emission at 541 nm of ErNPs and ECPM. i) Singlet oxygen generation under 980 nm laser excitation.

**Figure** **3**a,b shows typical excitation and emission pathways of ErNPs under 808 and 980 nm laser excitation. The emission peaks centered at 525, 541, and 657 nm could be attributed to transitions from the ^4^F_9/2_, ^4^S_3/2_, and ^2^H_11/2_ excited states to the ground state of ^4^I_15/2_, respectively. Under laser excitation, the ErNPs could offer bright downconversion luminescence (DCL) emission centered around 1550 nm, which was ascribed to the transition from ^4^ *I*_13/2_ to ^4^ *I*_15/2_ (Figure 3c). Interestingly, both the upconversion luminescence (UCL) and DCL emission intensities excited under 980 nm laser were stronger than those excited under 808 nm laser, which was mainly due to the stronger absorption around 980 nm of Er^3+^ ion (Figure S6, Supporting Information). Inspired by the outstanding UCL and DCL emissions of Er‐based rare earth nanomaterials, we further investigated whether the UCL from the ErNPs could excite the CPM (Figure 3d). As shown in Figure 3e, intense green emission from the ErNPs overlapped well with the absorption peak of CPM, indicating that the ErNPs could be potentially used for CPM activation.^[^ ^13b^ ^]^ The CPM was able to harvest the low‐energy photons in NIR range and emit green (541 nm) and red (657 nm) light, which could be absorbed by the inter CPM through fluorescence resonance energy transfer (FRET) (Figure 3e–h). The FRET process was confirmed by the strong depression of the UCL emission bands in the ECPM (Figure 3f). Time‐resolved photoluminescence measurements were further carried out to provide more evidence for the FRET process. As shown in Figure 3h, the UCL decay curves of ECPM revealed a significant decrease in the lifetime of Er^3+^ emission at 541 nm (from 86.4 to 46.1 *µ*s). The results revealed that the energy transfer efficiency from the ErNPs to inter CPM could be calculated (1−*τ*_DA_/*τ*_D_) to be ≈46.6%. Excitingly, the DCL emission of ECPM was independent of the integration of CPM, indicating that the ECPM could be acted as a stable NIR‐II image probe (Figure 3g). Due to the efficient FRET process in ECPM nanohybrids upon NIR excitation, it was facile to make the ECPM a NIR‐light excited ROS generator. Basically, we used commercially available singlet oxygen sensor green (SOSG) indicator to investigate the NIR‐triggered singlet oxygen (^1^O_2_) generation from CPM (Figure 3i). In the aqueous solution, the fluorescence of SOSG gradually increased with 980 laser irradiation time, which endowed the ECPM as efficient ROS generators. In addition, the ^1^O_2_ quantum yield (𝜑_∆_) was calculated to be 0.40 for ECPM by using methylene blue (MB) as the standard (Figure S7, Supporting Information).

### 2.2 DNA‐Mediated Self‐Assembly of ECPM

**Figure 4:**
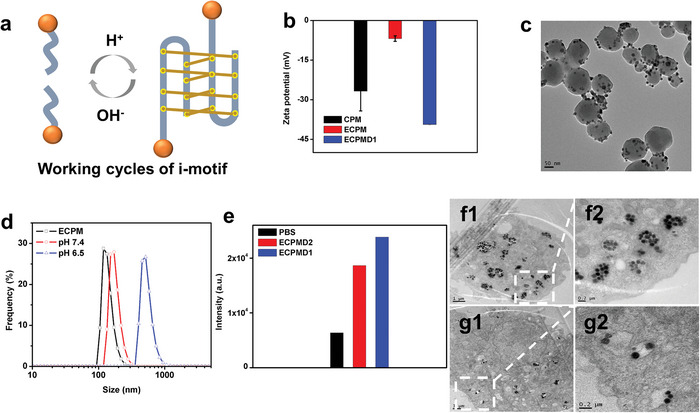
a) Scheme of the working cycles of i‐motif DNA (DNA1) strands modified ECPM (sphere: ECPM; line: i‐motif strands). The i‐motif DNA strands could intermolecularly form quadruplexes structures at acidic solutions, leading to self‐assembly of the ECPM into larger clusters. b) Zeta potential of the ECPM before and after DNA1 modification. c) TEM images of assembled ECPMD1 at pH 6.5. d) The DLS of ECPMD1 at pH 6.5 and 7.4 buffer solutions, the bare ECPM were used as control. e) Flow cytometric analysis of U87MG cellular uptake after treatment with the DNA1 or DNA2 modified ECPM (10 µg mL^−1^) for 1 h. f1) Bio‐TEM images of DNA1 modified ECPM in U87MG cells and f2) enlarged image of the dotted area. g1) DNA2 modified ECPM in cells and g2) enlarged image of the dotted area.

The tumor microenvironment generally exhibits several physiological alterations including lowered pH, elevated redox potential and overexpressed enzymes, which have been widely used as endogenous stimuli to control the behaviors of therapeutic agents.^[^ ^16^ ^]^ The extracellular tumor area has lower pH value (pH ≈ 6.5), compared to the normal blood or healthy tissues (pH ≈ 7.4).^[^ ^17^ ^]^ Also, the pH value continues to drop in the intracellular organelles, such as endosome or lysosome (pH ≈ 5.5 or less).^[^ ^18^ ^]^ In order to achieve stimuli‐responsive ECPM within the acidic tumor microenvironment, pH‐responsive DNA linkers, which contain cytosine‐rich “i‐motif” domains (i‐motif DNA, denoted as DNA1) were applied to conjugate with ECPM (**Figure** **4**a).^[^ ^4^, ^19^ ^]^ Thanks to the presence of unsaturated Zr coordination sites in the CPM, the phosphate‐modified oligonucleotides could be easily conjugated with the ECPM through the Zr–phosphate coordination interactions.^[^ ^13^, ^20^ ^]^ The TEM image showed no morphology changes for the ECPM after DNA conjugation (Figure S8, Supporting Information). As seen in Figure S9 (Supporting Information), the dynamic light scattering (DLS) measurements revealed that the hydrodynamic size of ECPM changed from ≈134.2 to ≈167.3 nm after DNA conjugation. The zeta potential measurements in Figure 4b showed that the surface charge of ECPM changed from −6.9 to −39.4 mV after surface functionalization with single DNA1 strands. These above results indicated that the DNA strands were successful conjugated on the surface of ECPM. Figure 4a shows the working cycles of the i‐motif DNA strand conjugated ECPM system, in which the ECPM NPs were modified with single DNA1 strand. At pH 7.4, DNA1 on ECPM surface was in random coils, ensuring ECPM NPs in the dispersed form due to the electrostatic repulsion interactions between the charged surfaces (Figure S10, Supporting Information). While at lower pH value, the hemiprotonation of cytosine‐cytosine base pairs intermolecularly transformed to quadruplex structures, leading to the self‐assembly of ECPM into clusters (Figure 4c).^[^ ^4^, ^19^, ^21^ ^]^ Interestingly, the average hydrodynamic diameter of DNA1‐ECPM (ECPMD1) in neutral buffer (pH 7.4) was ≈167.3 nm, which was increased to ≈509.2 nm when exposed to solutions at pH 6.5 (Figure 4d). As the acidity of the solution further increased, the size of assembled ECPM clusters continued to grow and reached ≈1072.9 nm at pH 4.0 (Figure S11, Supporting Information). The stabilities of the i‐motif DNA in 10% and 20% fetal bovine serum (FBS) were analyzed by agarose gel (Figure S12, Supporting Information). The results indicated that the DNA sequences remained intact in FBS solution, demonstrating the high stability of the i‐motif DNA.^[^ ^22^ ^]^ In addition, the ECPMD1 could maintain the virus‐like morphology in a long time, indicating its good stability in solution (Figure S13, Supporting Information). To demonstrate that the ECPM assembly was specific to cytosine‐rich domains, the ECPM modified with random single DNA strands (DNA2) were prepared as a control. In contrast, the TEM images in Figure S8b,c (Supporting Information) showed that there was no significant change in the size of DNA2‐ECPM (ECPMD2) at either pH 7.4 or pH 6.5 buffer solutions. Taken together, the above results indicated that the i‐motif‐DNA based self‐assembly in response to the acidic conditions may provide great potential to facilitate the efficient accumulation of ECPM in the acidic tumor microenvironment.

### 2.3 Self‐Assembly of ECPM in Live Cells

Inspired by the pH‐responsive self‐assembly property of ECPMD1 in buffer solution, the cellular uptake of ECPMD1 in live U87MG cells was investigated. As expected, an increasing fluorescence signal was observed after the cells were incubated with ECPMD1, indicating the efficient cellular uptake of ECPMD1 (Figure S14, Supporting Information). In addition, the quantitative flow cytometric (FCM) analysis revealed that ECPMD1 had better cellular accumulation than that cultured with ECPMD2 (Figure 4e). The enhanced cellular uptake was further confirmed by bio‐TEM images. As shown in Figure 4f,g, large ECPM clusters assembled by ECPMD1 were found in the cells, while almost no aggregation clusters were observed in ECPMD2 treated cells. The statistical analysis showed that about 78.8% clusters were composed of more than three single nanoparticles, indicating the good pH‐responsive self‐assembly property of ECPMD1 in live cells. All these results demonstrated that the pH‐responsive DNA strand conjugated ECPM could be efficiently accumulated and self‐assembled into clusters in live cells.

**Figure 5:**
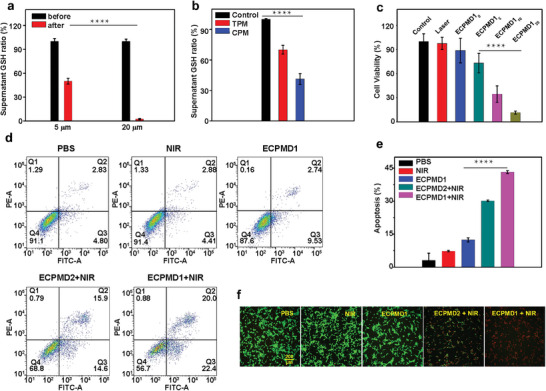
a) The GSH concentration in the supernatant after treatment with different amounts of CPM (*n* = 3). b) The GSH concentration in the supernatant treated with TPM and CPM (*n* = 3). c) Viability of the U87MG cells treated with ECPMD1 upon 980 nm laser irradiation (Subscript in ECPMD1_x_ (*x* = 0, 5, 10, 20 min) indicated the irradiation time) (*n* = 5). d) Flow cytometric analysis of the apoptosis in U87MG cells with indicated treatments. e) Quantitative analysis of the apoptosis in U87MG cells after treatments (laser irradiation for 5 min) (*n* = 5). f) Calcein AM/Propidium Iodide (PI) staining of live cells (green) and dead cells (red) with indicated treatments. Data are presented as means ± SD. ^*^ *p* < 0.05, ^**^ *p* < 0.01, ^***^ *p* < 0.001, ^****^ *p* < 0.0001, by one‐way ANOVA with Tukey's post hoc test were considered.

Inspired by the outstanding responsive accumulation performance in live cells, we hypothesize that such assembly strategy can also be used for improving the therapeutic effect. For this, we firstly tested the potential of Cu‐based CPM to deplete the intracellular GSH. Both zirconium‐based CPM and TPM with and without the Cu center were synthesized. Further characterization revealed that both types of photosensitizers exhibited the similar morphology and same crystal structure, both of which demonstrated strong absorption around 420 and 650 nm red fluorescence (Figures S15–S18, Supporting Information and Figure 2). To investigate the GSH depletion ability, both CPM and TPM were incubated with GSH solution over time. After that, the remaining GSH content in the supernatant was analyzed by a fluorometric thiol assay kit. Surprisingly, GSH content in the supernatant was significantly decreased when treated with CPM, with less than 3% left when the concentration of CPM was up to 25 µm (**Figure** **5**a). Further experiments showed the decrease of GSH level with the increasing concentration of CPM, demonstrating its efficient GSH depletion ability (Figure S19, Supporting Information). Moreover, much lower GSH levels were detected in solutions with CPM when compared to those with TPM (Figure 5b). It should be noted that TPM could also reduce certain amount of GSH, probably due to the nonspecific adsorption by the porous crystal structure. In addition, the GSH concentration variations in live U87MG cells were also detected by using GSH assay kit. As shown in Figure S20 (Supporting Information), lower GSH level was observed in the cells treated with ECPMD1 under laser irradiation, mainly due to the synergistic GSH consumption ability of the CPM and the produced ROS upon laser irradiation. The above results demonstrated that the CPM with the copper active center could act as an excellent GSH sacrificial agent.

The outstanding performance of NIR‐induced ROS generated by ECPM combined with its GSH scavenging effect prompted us to investigate its cell killing effect. As shown in the 3‐(4,5‐dimethylthiazol‐2‐yl)‐2,5‐diphenyltetrazolium bromide (MTT) assay, the ETPM had negligible cytotoxicity. The results showed low but detectable cytotoxicity on U87MG cells with ≈73.8% cell viability when the cells were treated with ECPM nanohybrids at higher concentration (100 µg mL^−1^) (Figure S21, Supporting Information), mainly due to the contribution of elevated ROS levels through the GSH‐depletion role of the Cu center in CPM.^[^ ^23^ ^]^ Moreover, the ECPM treated cells also showed decreased GSH level observed from the confocal images, owing to the GSH scavenging effect of the CPM (Figure S22, Supporting Information). However, the cellular level of GSH had no noticeable changes for the cells cultured with ETPM. In vitro experiments were further carried out to determine the possibility of the ECPM as a NIR‐induced photodynamic therapy (PDT) agent. As an efficient radical oxygen species (ROS) generator, the intracellular ROS generation was evaluated by using the ROS indicator 2′, 7′‐dichlorofluorescein diacetate (DCFH‐DA). As shown in Figure S23 (Supporting Information), stronger fluorescence signals were detected in the ECPM‐treated cells upon laser irradiation, implying the increased ROS species in cells. By contrast, ECPM without laser irradiation or other control groups induced almost no ROS generation. No obvious cell death was observed from the MTT results in Figure 5d even after 20 min laser irradiation. As expected, the ECPMD1 treated cells showed gradual increase in cell death with increasing irradiation time (Figure 5c), probably results from the GSH scavenging effect improved photodynamic effect. The flow cytometric analysis and Live/Dead staining images demonstrated analogous results (Figure 5d–f, Figure S24, Supporting Information). The control groups (phosphate buffered saline (PBS), NIR) led to negligible cytotoxicity. Without irradiation, ECPMD1 alone treatment also led to some cells (≈12.4%) to undergo apoptosis, indicating ECPMD1 alone exhibited anticancer ability ascribing to the GSH scavenging effect of CPM (Figure 5d). Of note, upon laser irradiation, the most effective anticancer ability was achieved after ECPMD1 treatment at a low concentration (10 µg mL^−1^) under a short time irradiation (5 min), verifying the combination of GSH scavenging effect and NIR‐induced photodynamic therapy (Figure 5e). As expected, the pH‐responsive DNA1 strand conjugated ECPM treatment demonstrated better performance in cell apoptosis (an increase of ≈13.1%) in comparison with the treatment of DNA2 strand conjugated one, mainly due to the enhanced cell internalization of the ECPMD1. Similarly, the Live/Dead staining images further verified the outstanding performance of the ECPMD1 treatment in cell apoptosis (Figure 5f, Figure S24, Supporting Information). Furthermore, the JC‐1 assay was utilized to study the loss of mitochondrial membrane potential (MMP), which could be used to evaluate the free radical‐caused cell damage. As shown in Figures S25 and S26 (Supporting Information), the of ECPMD1+NIR group displayed much weaker red fluorescence than that in the other treatment groups. The decrease in red/green fluorescence ratio suggested mitochondrial depolarization. The experimental results revealed that the U87MG cells treated with ECPMD1 upon NIR irradiation displayed obvious mitochondrial membrane potential loss. In addition, we examined the apoptosis‐involved factors caspase 3 (CASP3), bcl2 (BCL2), and bax (BAX) in ECPMD‐treated cells. In U87MG cells, ECPMD combined with NIR laser increased BAX level and reduced BCL2 level, leading to an increased BAX/BCL2 ratio (control: 1.00 ± 0.09, ECPMD1_NIR_: 2.39 ± 0.05, ECPMD2_NIR_: 2.29 ± 0.08), which reflected ongoing apoptosis of tumor cells (Figure S27, Supporting Information). Caspase‐3 levels of ECPMD1 and ECPMD2 groups were higher than those of the control groups. Also, PDT induced stress response could cause the upregulation of HSP90. Meanwhile, cells treated with ECPMD1 and ECPMD1 with light showed upregulation of HSP90 while DCPM1 and DCPM2 and light exposure alone had no effect. These data indicate that ECPMD1 and ECPMD1 combined with light induce a stress response within treated cells and subsequent upregulation of HSP90. Collectively, these results indicated that both pH‐responsive cell internalization and GSH scavenging effect of ECMPD1 could contribute to the amplify NIR‐induced photodynamic therapy in vitro.

### 2.4 In Vivo Assembly of ECPM and Its Therapeutic Effect

**Figure 6:**
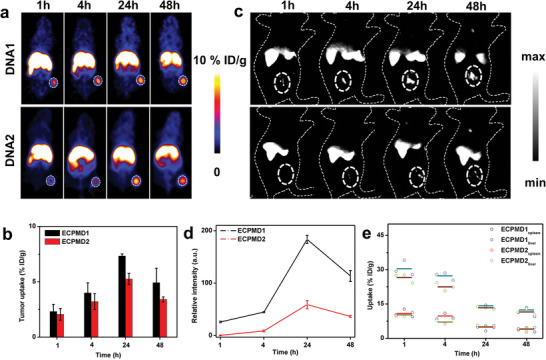
a) Representative positron emission tomography (PET) images of U87MG tumor‐bearing mice at 1, 4, 24, and 48 h postinjection (p.i.) of the ^64^Cu‐labeled ECPM with DNA1 and DNA2 modification. b) The corresponding PET quantification on tumor uptake of ^64^Cu labeled DNA1 and DNA2 conjugated ECPM (*n* = 3). c) In vivo NIR‐II imaging of the U87MG tumor‐bearing mice at 1, 4, 24, and 48 h p.i. (the tumor was located in the dotted circle. imaging details: 1300 nm long pass filter, 808 nm laser). d) Quantitative analysis of the NIR‐IIb signal intensity in the tumor area by ImageJ software. e) Spleen and liver uptakes of ECPMD NPs over times. Data are presented as means ± SD.

To investigate whether the acidic microenvironment‐driven self‐assembly of ECPM would contribute to the enhanced tumor accumulation in vivo, ^64^Cu‐labeled ECPM with DNA1 and DNA2 strand modification were intravenously injected into U87MG tumor‐bearing mice and the biodistribution of the ECPM was monitored by positron emission tomography (PET) imaging. The tumor, liver and spleen uptakes of ECPM were calculated by region of interest (ROI) analysis of the PET images. As shown in **Figure** **6**a, the ECPM could quickly accumulate at the U87MG tumor site within 1 h, and the tumor uptake gradually increased, reaching up to ≈7.2%ID g^−1^ at 24 h postinjection (p. i.) (Figure 6b). Such ECMPD showed a relatively long blood circulation, consistent with the PET image data (Figure S28, Supporting Information). It is believed that the accumulation at tumor site was mainly derived from the enhanced permeability and retention (EPR) effect.^[^ ^24^ ^]^ Compared to ECPMD2, more ECPMD1 was accumulated at the tumor site at each corresponding time point (Figure 6b). Interestingly, the ECPMD1 to ECPMD2 uptake ratio (denoted as DNA1/DNA2) gradually increased over time, which can be ascribed to the tumor microenvironment induced nanoparticle self‐assembly with prolonged tumor retention (Figure S29, Supporting Information). NIR‐IIb (1500–1700 nm) imaging is one of the most promising techniques for in vivo monitoring, thanks to the advantages of deep imaging depth, reduced scattering and high spatial resolution.^[^ ^25^ ^]^ Since ErNPs exhibited strong NIR‐IIb window emission (≈1550 nm) under 808 nm laser irradiation (the 808 nm laser could cause less phototoxicity, so the 808 nm instead of 980 nm laser was used for the in vivo imaging), we believed that this property could be used for tracking the ECPM in vivo. As shown in Figure 6c, the tumor tissue from the group with ECPMD1 treatment showed a gradual increase in NIR‐IIb fluorescence signals from 1 to 48 h p.i (Figure 6d), which was in accordance with the PET results. In contrast, the NIR‐IIb tumor signal from ECPMD2 treated group was much weaker, suggesting that the DNA‐mediated nanoparticle assemblies greatly prolonged the tumor retention, along with enhanced tumor imaging capacity. As shown in Figure 6e, the liver and spleen uptakes of ECMP gradually decreased over time, suggesting the ECPM NPs were mainly excreted through the reticuloendothelial system, which is typical for inorganic NPs.^[^ ^26^ ^]^ In addition, the ECPM could be easily cleared from the major organs over time (Figures S30 and S31, Supporting Information).

**Figure 7:**
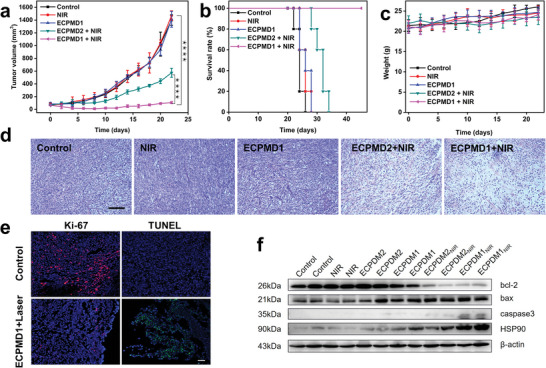
a) Tumor growth curves of mice bearing U87MG tumors subjected to indicated treatments (*n* = 5 mice per group). b) Mouse survival rates after various treatments of PBS, NIR, ECPMD1, ECPMD2 + NIR and ECPMD1 + NIR using Kaplan–Meier analysis followed by a log‐rank test. Mean ± s.d. (*n* = 5 mice per group). c) Body weight changes of the mice with various treatments for 22 days. d) Representative H&E staining images of tumor sections after various treatments. Scale bars, 100 µm. e) Ki‐67 and TUNEL staining merge images of the tumor sections after various treatments at 24 h p.i., scale bar: 50 µm. f) Western blot (WB) analysis of proteins obtained from the U87MG tumor after different treatments. Data are presented as means ± SD. Tumor growth curves were assessed by two‐way ANOVA with Turkey's testing for multiple comparisons; the other data were assessed by one‐way ANOVA with Tukey's post hoc test. ^*^ *p* < 0.05, ^**^ *p* < 0.01, ^***^ *p* < 0.001, ^****^ *p* < 0.0001.

Finally, we assessed the potential photodynamic effect by intravenous injection of ECPM into U87MG tumor mice (*n* = 5 per group). The mice were treated with 980 laser irradiation (0.5 W cm^−2^) for 20 min at 24 h p.i. As shown in **Figure** **7**a, negligible inhibition of tumor growth was observed in the PBS and PBS plus laser irradiation groups. The ECPMD1 alone group slightly delayed the tumor growth, implying that GSH depletion induced some tumor inhibitory effect. In contrast, the ECPMD2 + NIR treated group presented obvious delay of tumor growth, which was ascribed to the synergistic GSH depletion/PDT effect. The GSH depletion ability of CPM in ECPMD2 + NIR and ECPMD1 + NIR groups was also observed in the tumor tissues (Figure S32, Supporting Information). Compared with the control and other treatment groups, the GSH concentration in the tumor tissue with ECPMD1 + NIR treatment was the lowest, which could be ascribed to the synergistic depletion effect of CPM alone and the generated ROS under laser irradiation. Different from DNA2 modified ECPM, the antitumor efficacy was further enhanced by the treatment of ECPMD1 + NIR, clearly indicating the assembly strategy provided additional benefit of tumor inhibition. It is not surprising that the survival rate of mice treated with ECPMD1 + NIR was much higher compared to the other groups (Figure 7b). In addition, there were negligible body weight changes throughout the experimental period, suggesting relatively high biocompatibility and biosafety of these materials (Figure 7c). To better understand the efficacy of the treatments, the tumors were harvested for histological analysis at 24 h after the treatments. Hematoxylin and eosin (H&E) staining images indicated severe structure disruption in the ECPM treated groups, especially in the DNA1 modified ECPM group (Figure 7d). The proliferative capacity of the tumor was evaluated by Ki‐67 immunohistochemistry assays (Figure S33, Supporting Information). As shown in Figure  **7**e, the obvious apoptosis and significantly inhibited proliferation of the tumor cells were observed in the ECPMD1 + NIR group. Terminal deoxynucleotidyl transferase mediated dUTP‐biotin nick and labeling (TUNEL) staining images revealed similar results (Figure S34, Supporting Information). Furthermore, we examined the CASP3, BCL2, BAX, and HSP90 in tumor tissues (Figure 7f). Also, the BAX/BCL2 ratio after treatment with ECPMD1_NIR_ and ECPMD2_NIR_ increased from 1.01 to 7.02 and 1.01 to 6.58, respectively (Figure S35, Supporting Information). Meanwhile, the levels of CASP3 and HSP90 were elevated in presence of ECPMD and NIR in tumor tissues, which is consistent with the results in U87MG cells. After irradiation the effect of ECPMD1 is even more prominent. Furthermore, histological analysis was carried out on major organs of mice after the PDT treatment, which showed no apparent pathological abnormalities compared with those in the normal mice (Figure S36, Supporting Information). The blood biochemical analysis was performed to evaluate the biosafety of ECPM. As shown in Figure S37 (Supporting Information), both the liver function indices of alanine aminotransferase (ALT) and alkaline phosphatase (ALP), and the kidney function indices of blood urea nitrogen (BUN), phosphorus (P), and creatinine (CRE) were found to have little variation compared with the that in the control groups, the changes of Ca, glucose (GLU), cholesterol (CHOL), total bilirubin (TBIL), amylase (AMY), and creatine kinase (CK) were all negligible in the treatment groups, implying the biocompatibility of the ECPM. Additionally, the blood routine assay tests further verified that the ECPM treatment had little to no toxicity to the mice (Figure S38, Supporting Information). Taken together, all these results showed that the ECPM held great promise for tumor microenvironment responsive self‐assembly with desired NIR‐induced outcome synergistically with GSH scavenging effect.

## 3 Conclusion

In this work, we developed a pH‐responsive self‐assembly strategy and further investigated the promising capability in NIR‐IIb imaging and cancer therapy. The acidic tumor microenvironment can effectively trigger the self‐assembly of i‐motif DNA modified ECPM both in vitro and in vivo, thus improving the nanoparticle accumulation and amplifying the NIR‐II fluorescence signals and PDT effect upon NIR light illumination. Overall, such in situ nanoparticle assembly strategy provides a promising solution for enhanced tumor targeting and therapeutic efficacy in a controllable manner. As the saying goes, “Unity makes strength.” We expect this concept would facilitate the development of novel biohybrids by controllable assembly for emerging bioapplications.

### Statistical Analysis

Unless otherwise noted, all data are represented as the mean value ± standard deviation (SD) from at least 3 independent experiments (*n* = 3). The significant differences in the experimental data were evaluated using one‐way ANOVA with Tukey's significant difference post hoc test (using Graphpad software). The calculated probability (*p*) was distinguished as ^*^ *p* < 0.05, ^**^ *p* < 0.01, ^***^ *p* < 0.001, ^****^ *p* < 0.0001. Detailed processing of data for each analysis is described in each figure legend.

## Conflict of Interest

The authors declare no conflict of interest.

## Author Contributions

L.H. and N.Z. contributed equally to this work. L.H., J.M., S.L., and X.C. designed this project. L.H., N.Z., S.W., and J.M. conducted the in vitro and in vivo experiments. Q.W., J.D., and G.C. run the optical experiments and interpreted the results. Z.W. carried out the PET study. Z.C. did the western blot experiments. The manuscript was written through contributions of all authors. All authors have given approval to the final version of the manuscript.

## Supporting information

Supporting Information

## Data Availability

The data that support the findings of this study are available in the supplementary material of this article.
