# Supplementary material for: Responsive Accumulation of Nanohybrids to Boost NIR‐Phototheranostics for Specific Tumor Imaging and Glutathione Depletion‐Enhanced Synergistic Therapy
Source: Adv Sci (Weinh). 2022 Nov 14;10(1):2205208. doi: 10.1002/advs.202205208 (PMC9811476; doi:10.1002/advs.202205208)
Supplement: Supplementary file 1 — Supporting Information [file ADVS-10-2205208-s001.pdf]

ADVANCED SCIENCE
Open Access

Supporting Information

for *Adv. Sci.*, DOI 10.1002/advs.202205208

Responsive Accumulation of Nanohybrids to Boost NIR-Phototheranostics for Specific Tumor Imaging and Glutathione Depletion-Enhanced Synergistic Therapy

Liangcan He, Nannan Zheng, Qinghui Wang, Jiarui Du, Shumin Wang, Zhiyue Cao, Zhantong Wang, Guanying Chen, Jing Mu*, Shaoqin Liu* and Xiaoyuan Chen**

Supporting Information

Responsive Accumulation of Nanohybrids to Boost NIR-phototheranostics for Specific Tumor Imaging and Glutathione Depletion-Enhanced Synergistic Therapy

Liangcan He, Nannan Zheng, Qinghui Wang, Jiarui Du, Shumin Wang, Zhiyue Cao, Zhantong Wang, Guanying Chen, Jing Mu*, Shaoqin Liu*, Xiaoyuan Chen**

1. General Information

Materials. Tetrakis(4-carboxyphenyl) porphyrin, polyvinylpyrrolidone (MW 40000) and singlet oxygen sensor green (SOSG) were purchased from VWR international. Gadolinium(III) chloride hexahydrate ($GdCl_3 \cdot 6H_2O$), erbium(III) chloride hexahydrate ($ErCl_3 \cdot 6H_2O$), oleic acid, octadecene, ammonium fluoride, sodium hydroxide, cyclohexane, zirconyl chloride octahydrate, benzoic acid, methyl thiazolyl tetrazolium (MTT), $CuCl_2 \cdot 2H_2O$, COOH-PEG (2000), 2′, 7′-dichlorofluorescein diacetate (DCFH-DA) and N, N-dimethylformamide were purchased from Sigma-Aldrich and Macklin. The DNA was purchased from Integrated DNA technologies and Sangon Biotech. All starting materials and solvents, unless otherwise noted, were purchased from commercial sources and used without further purification.

Characterization. Transmission electron microscopy (TEM) images were acquired on the FEI Tecnai T12 electron microscope. Elemental mapping was acquired on the scanning transmission electron microscope (JEM-3200FS, JEOL, Japan). The hydrodynamic diameter distribution and zeta potential of various MOF NPs were measured on a scientific dynamic light scattering nanoparticle analyzer (SZ-100, Horiba). The XRD pattern was recorded on a D8 Advance diffractometer (Bruker, Germany). UV-Vis absorption spectrum was recorded on a Shimadzu UV-2501 spectrophotometer. Confocal laser scanning microscopy (CLSM) images was recorded on a Zeiss LSM 780 microscope. Flow cytometry analysis was carried out on a BD Beckman Coulter flow cytometer (Brea, CA). H&E tissue and cell staining was performed by BBC Biochemical (Mount Vernon, WA) and the images were collected using a BX41 bright field microscopy (Olympus). PET images were recorded on an Inveon small-animal PET scanner system (Siemens Medical Solutions, USA). NIR-II images were acquired on home-made equipment.[1-3]

2. Experimental Section

Materials. Tetrakis(4-carboxyphenyl) porphyrin, Cu (II) meso-tetra(4-carboxyphenyl) porphine, polyvinylpyrrolidone (MW 40000) and singlet oxygen sensor green (SOSG) were purchased from VWR international. Gadolinium (III) chloride hexahydrate ($GdCl_3{\cdot}6H_2O$), erbium(III) chloride hexahydrate ($ErCl_3{\cdot}6H_2O$), oleic acid, octadecene, ammonium fluoride, sodium hydroxide, cyclohexane, zirconyl chloride octahydrate, benzoic acid, methyl thiazolyl tetrazolium (MTT), COOH-PEG (2000), 2′, 7′-dichlorofluorescein diacetate (DCFH-DA) and N, N-dimethylformamide were purchased from Sigma-Aldrich and Macklin. The DNA was purchased from Integrated DNA technologies and Sangon Biotech.

CPM synthesis. Tetrakis(4-carboxyphenyl) porphyrin (5.0 mg), Cu(II) meso-tetra(4-carboxyphenyl) porphine (5. 0 mg), zirconyl chloride octahydrate (30.0 mg), and benzoic acid (280 mg) were mixed in 10 mL N, N-dimethylformamide (DMF) solution. The reaction mixture was heated at 90 °C for 5-7 h. After the reaction was done, the product was collected by centrifugation at 12 000 rpm for 10 min followed by washing with fresh DMF three times, which was then re-dispersed in 10 mL of DMF for further use.

Synthesis of core $NaGdF_4$:10%Er nanoparticles. The small hexagonal phase (β-) NaGdF4:10%Er nanoparticles were synthesized following a previously reported method.[4] In a typical procedure, $GdCl_3 \cdot 6H_2O$ (334.53 mg, 0.9 mmol), $ErCl_3 \cdot 6H_2O$ (38.17 mg, 0.1 mmol), oleic acid (OA, 6.0 mL) and octadecene (ODE, 15.0 mL) were mixed together and heated to 160 °C with vigorous stirring under argon for 60 min to form a homogeneous transparent solution, and then solution was cooled down to room temperature. A methanol solution (10.0 mL) of ammonium fluoride (0.148 g, 4 mmol) and sodium hydroxide (0.1 g, 2.5 mmol) was added drop by drop and stirred for 1 h. The solution was then slowly heated and degassed at 110 °C for 20 min and then refilled with argon and degassed three times in total. After that the solution was heated to 280 °C within 10 min and reacted for 60 min under argon. After the solution was cooled back to room temperature, the products were precipitated from the solution with ethanol and washed with ethanol and water (1:1) three times. The nanoparticles were finally dispersed in 10 mL of cyclohexane for further use.

Synthesis of core-shell Er NPs ($NaGdF_4$:10%Er@$NaGdF_4$). In a typical procedure, $GdCl_3 \cdot 6H_2O$ (148.68 mg, 0.4 mmol, oleic acid (OA, 4.0 mL) and octadecene (ODE, 6.0 mL) were mixed together and heated to 140 °C with vigorous stirring under argon for 60 min to form a homogeneous transparent solution, and then solution was cooled down to room temperature. Then the above core nanoparticles solution was injected. After that, a methanol

solution (10.0 mL) of ammonium fluoride (59.2 mg, 1.6 mmol) and sodium hydroxide (40 mg, 1.0 mmol) was added drop by drop and stirred for 1 h. The solution was slowly heated and degassed at 100 °C for 20 min and then refilled with argon and degassed three times in total. After that the solution was heated to 280 °C within 10 min and reacted for 60 min under argon. After the solution was cooled back to room temperature, the products were precipitated from the solution with ethanol and washed with ethanol and water (1:1) three times. The nanoparticles were finally dispersed in 10 mL of cyclohexane for further use.

Ligand exchange of ErNPs. 1 mL of 7.2 mg ErNPs was added into 15 mL of chloroform followed by adding 1 g of PVP (M.W. 40 K). The above mixture was then stirred for 24 hours. Then the products were purified and washed with ethanol three times and redispersed in 500 μL ethanol for further use.

Preparation of ECPM. Typically, 1 mL of CPM (1 mg/mL) in DMF was mixed with 2 mL fresh DMF solution. 400 μL of ErNPs was then added into the above solution drop by drop. After stirring for 24 hours, the samples were purified and redispersed in water

DNA modification of ECPM. The CPM were conjugated with DNA strands based on previous reported method with minor modification.[5] Typically, the 100 μL 500 μM phosphate-terminated DNA single strand were mixed with 500 μL CPM/ErNPs in HEPES buffer solution for overnight**.** Then, NaCl solution was added to get the final salt concentration of 50 mM and reaction continued for another few hours. Finally, the products were purified and stored for further use. DNA1: 5'-phos-TTAACAAATTATATTATTCCCCTTTTCCCC-3', DNA2: 5'-phos-TTAACA AATTATATTATTAAAAAAAAAAAA-3'.

Singlet oxygen generation test. SOSG was used for detecting the produced singlet oxygen. Before laser irradiation, 1 μL 5 mM SOSG dimethyl sulfoxide (DMSO) solution was added to the test

solutions. The samples were then irradiated with 980 nm laser light for 0, 2, 4, 6, and 8 min. Every 2 min, the fluorescence intensity of the solution was measured by a fluorimeter.

Quantum yields mesurement. In order to quantify the quantum yields of the CPM and ECPM hybrid system, the 1,3-diphenylisobenzofuran (DPBF) is used as the singlet oxygen ($^1O_2$) indicator to evaluate the $^1O_2$ generation. The $^1O_2$ quantum yield (φ_Δ) is calculated through monitoring the oxidation of DPBF with a UV-vis spectrophotometer. Herein, the relative quantum yield is determined by a standard method. For this, methylene blue (MB, φ_Δ(std)=0.52) is used as the standard to determine the $^1O_2$ quantum yields (*J. Am. Chem. Soc.*, 2013, 135, 18850; *Chem. Commun.*, 2015, 51, 10831). As both of the MB and porphyrin MOFs exhibit absorption around 660 nm, 660 nm laser is used for the tests. Briefly, an oxygen-saturated solution of CPM, ECPM, and MB individually containing 60 µM DPBF was prepared (in the dark) and irradiation with 660 nm laser (20 mW/cm^2). The absorption of DPBF was monitored at different time points. And the quantum yields were calculated by the following equation.

$$\varphi_\Delta = \varphi_{\Delta(\mathrm{std})} \times \frac{S_x}{S_{std}} \times \frac{F_{std}}{F_x}$$

Where,

x: sample (MB, CPM, ECPM);

std: MB;

S: slope of the absorbance of DPBF (418 nm) vs irradiation time;

F: absorption correction factor, $F = 1 - 10^{-OD}$ (OD: the optical density of the sample and MB at 660 nm).

Study Approval

The procedures for the animal experiments were implemented under protocols of the National Regulation of China for Care and Use of Laboratory Animals. All animal experiments were carried out in accordance with the Guide Protocol of Laboratory Animals, approved by the animal care and use committee (ACUC) of the National Institutes of Health Clinical Center and Harbin Institute of Technology under the production license number SCXK(BJ)2021-0011 and use license number SYXK(HLJ)2018-0003.

Cell Culture. The U87MG cells were cultured at 37 ^oC and with 5% CO_2 in DMEM containing 10% fetal bovine serum and 1% penicillin/streptomycin.

Cytotoxicity assay (MTT). The cell viability of U87MG cells was determined by MTT assay. U87MG cells were seeded into a 96-well plate at 10^4/well density and incubated for 24h at 37 ^oC under 5% CO_2. Then, the CPM, ECPM dispersed in DMEM were added to each well to give final particle concentrations of 0, 0.8, 1.6, 3.2, 6.4, 12.5, 25.0, 50 and 100 μg/mL. The cells were incubated for another 24 h at 37 °C under 5% CO_2. After incubation, the cells in the plate were washed using PBS to remove the non-uptake particles, and 100 μL fresh medium was added. Then 20 μL of sterilized MTT reagent (5 mg/mL in 1x PBS) was added to each well, and the plates were further incubated at 37 °C for 4 h. After incubation, medium was removed, and the precipitated formazan crystals were dissolved by adding DMSO. For the PDT experiments, 10 μg/mL was used as the concentration of ECPM. The absorption was measured using a microplate reader. All the samples were prepared in triplicate.

NIR-induced photodynamic test. U87MG cells were incubated with CPM, ECPM at a final concentration of 10 μg/mL for 24 h, excessive NPs were then removed by PBS washing, and 100 μL of fresh medium was added. Then, 980 nm laser (500 mW/cm^{-2}) was used to irradiate each cell well in the 96-well plate for 0, 5, 10 or 20 min and then replaced with fresh culture medium for another 12 h at 37 °C. Cell viability was determined by MTT assay. All the samples were prepared in triplicate.

Flow cytometry and fluorescence imaging study. The cellular uptake of CPM, ECPM was evaluated in U87MG cells. The cells were seeded in a 12-well plate at 3×10^5 cells per well and further cultured for 12 h. The nanoparticles were added to the wells at a final concentration of 10 μg/mL. After incubation for 1, 2, 4 and 8 h, the medium was removed, and the cells were washed with PBS. Then cells were collected and used for the flow cytometry studies. For the fluorescence imaging study, the nanoparticles (10 μg/mL) were added into the cells in 8-well detachable chambers. After different treatments, the medium was removed, and the cells were washed with PBS. Then images were observed under a Zeiss LSM 780 confocal microscope.

***In vivo* PET imaging.** The ^{64}Cu-labeled CPM/ECPM were systematically administrated into U87MG tumor-bearing mice at around 100 μCi. The PET images were collected on a micro Inveon PET scanner at 1, 4, 24, and 48 h p.i. Regions of interest (ROIs) were circled, and the corresponding radioactivities were quantified on the areas of tumors, spleens and livers in the decay-corrected whole-body coronal images.

NIR-II imaging. Typically, the imaging agents were firstly injected intravenously. Then, the mice were anesthetized using isoflurane. For the imaging experiment, all NIR-II images were collected on a two-dimensional InGaAs array (Princeton Instruments) based on a home-made set-up. The excitation laser was an 808 nm laser set up at a power density of ~0.15 W/cm^2. Emission was typically collected with 1300 nm long pass filter. A variable exposure time was used for the InGaAs camera to capture images in the NIR-II window.

GSH concentration in cells after various treatments. The intracellular consumption of GSH was detected by GSH assay kit. Briefly, U87 cells were seeded into 6-well plates for 24 h (37 °C, 5% CO_2). Then, the cells were treated as following: (1) Control, (2) NIR, (3) ECPMD1, (4) ECPMD2+NIR and (5) ECPMD1+NIR. After 24 h incubation, group (4) and (5) were irradiated by NIR. 12 h later, the treated U87 cells were washed three times by PBS and collected for detection. Then, the obtained cells

suspended in 1 mL PBS were crushed by an ultrasound cell crusher. Finally, the intracellular GSH content was detected by the Reduced GSH Assay Kit (Nanjing Jiancheng Bioengineering Institute, A006-2-1).

GSH concentration in tumor after various treatments. The tumoral GSH depletion was evaluated when tumor size reached 300 mm^3. The mice were randomly divided into six groups: (1) Control, (2) NIR, (3) ECPMD1, (4) ECPMD2+NIR and (5) ECPMD1+NIR. The formulations were administrated by intratumoral injection. At 12 hours after injection, group (4) and (5) were irradiated by NIR. Then the tumors were harvested, the tumor tissues were frozen in liquid nitrogen and then pulverized. Subsequently, the proteins in the sample were removed by the buffer, followed by homogenate treatment. Finally, the intracellular GSH content was detected by the Reduced GSH Assay Kit (Nanjing Jiancheng Bioengineering Institute, A006-2-1) according to reported method.[6]

Western Blotting Analysis. The expression level of Bcl-2, Bax, caspase 3 and HSP 90 proteins were analyzed by Western blotting. Briefly, U87 cells were seeded into 6-well plates for 24 h (37 °C, 5% CO_2). Then, the cells were treated as following: (1) Control, (2) NIR, (3) ECPMD1, (4) ECPMD1, (5) ECPMD2+NIR and (6) ECPMD1+NIR. After 24 h incubation, group (4) and (5) were irradiated by NIR. 12 h later, the treated U87 cells were was lysed in lysis buffer. Following centrifugation at 12000 rpm at 4 °C for 10 min. A total of 20 μg of protein per lane was run on sodium dodecyl sulphate-polyacrylamide gel electrophoresis and then transferred onto onto polyvinylidene-difluoride (PVDF) membranes for western blotting. The membranes were blocked with 5% milk at room temperature for 1h and then incubated with primary antibodies overnight at 4 °C. The membranes were washed and further incubated with a secondary antibody (appropriately diluted) at room temperature for 1h, and then detected using the Pierce ECL Western Blotting Substrate (Thermo Fisher Scientific). β-actin was used as the control. ImageJ was used to acquire images. Cumulative densitometric analyses of the western blotting images were performed by Image J.

***In vivo* anticancer effect.** When the tumor size reached 60-100 mm^3, U87MG tumor bearing mice were randomly divided into five groups and treated with various formulations: (1) PBS, (2) laser, (3) ECPM, (4) ECPM with DNA-2 strand modification + laser, (5) ECPM with DNA-1 strand modification + laser. ECPM were injected intravenously at a concentration of 25 mg/kg. In the experiments, the 980 nm laser (0.5 W/cm^2, 20 min) was used for the irradiation of tumors at 24 h post-injection. Two days later, another injection and treatment were applied with the same parameters. The tumor sizes were measured with a caliper every two days and calculated by the formula (width × width × length)/2. Body weight of each mice was monitored every two days. Tumors and major organs were sectioned for hematoxylin-eosin staining (H&E) and immunofluorescence analyses.

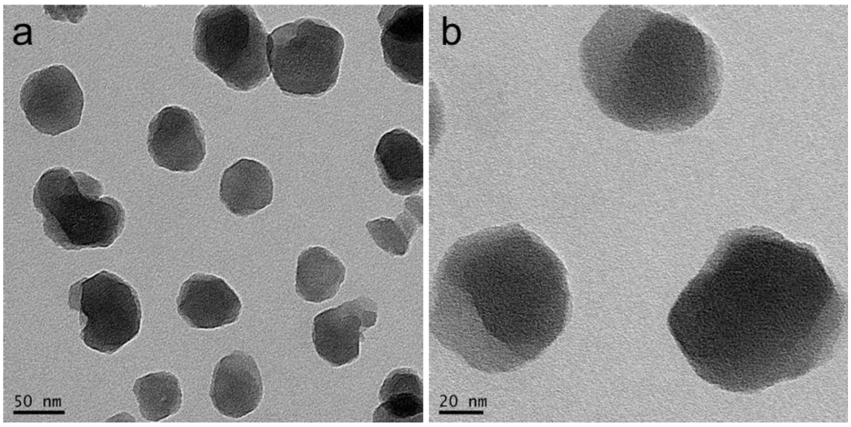

Figure S1. TEM images of CPM at (a) low and (b) high magnifications.

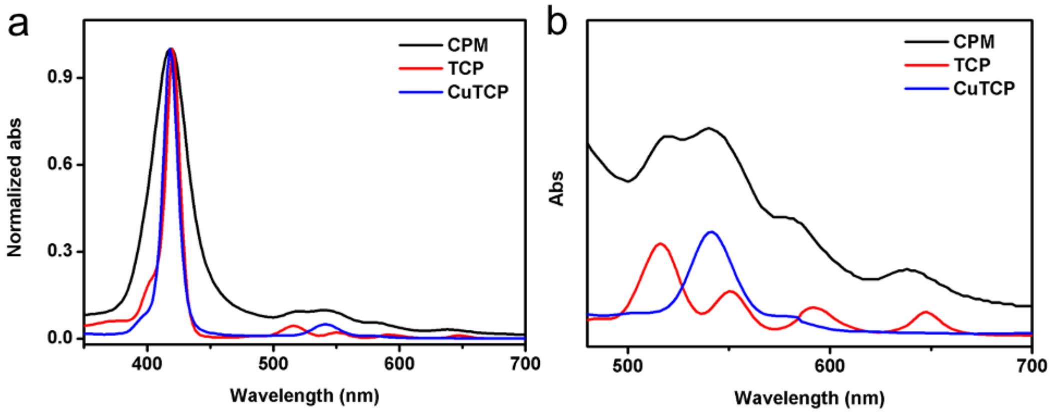

Figure S2. (a) UV-vis spectra of CPM, TCP, and CuTCP, (b) and the corresponding enlarged image in the region of 480-700 nm.

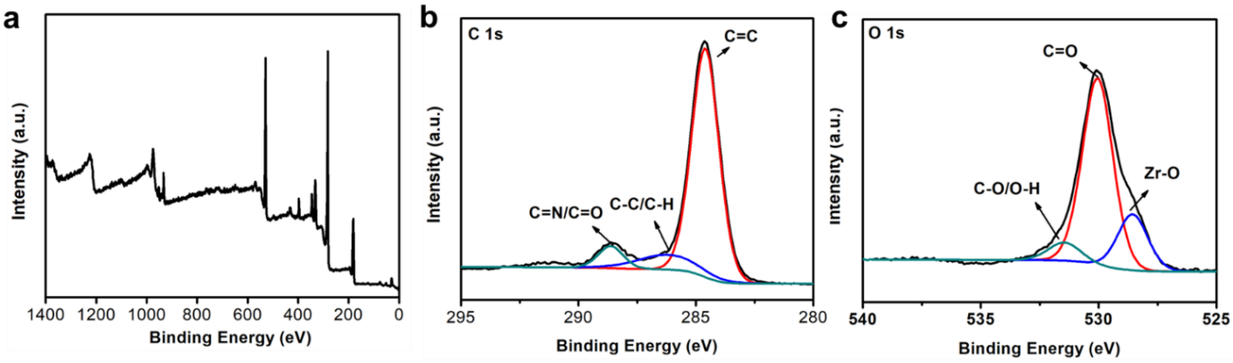

Figure S3. High-resolution XPS of (a) CPM, (b) C 1s and (c) O 1s.

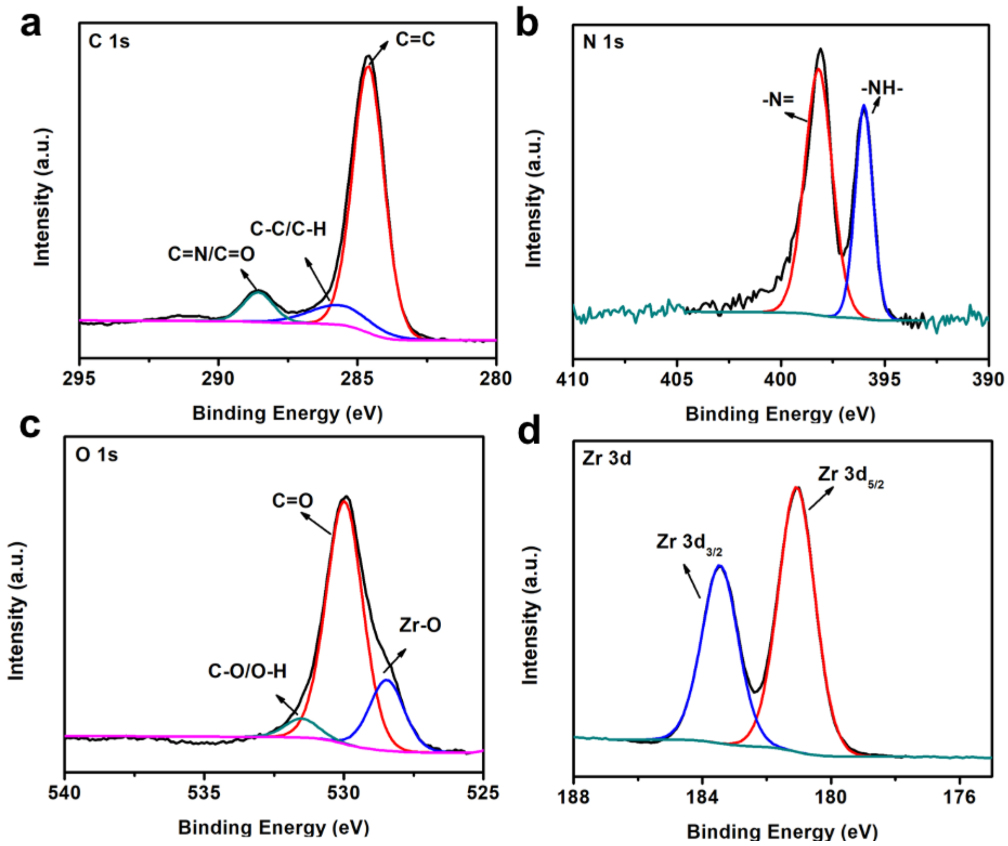

Figure S4. High-resolution XPS of (a) C 1s, (b) N 1s, (c) O 1s and (d) Zr 3d for TPM.

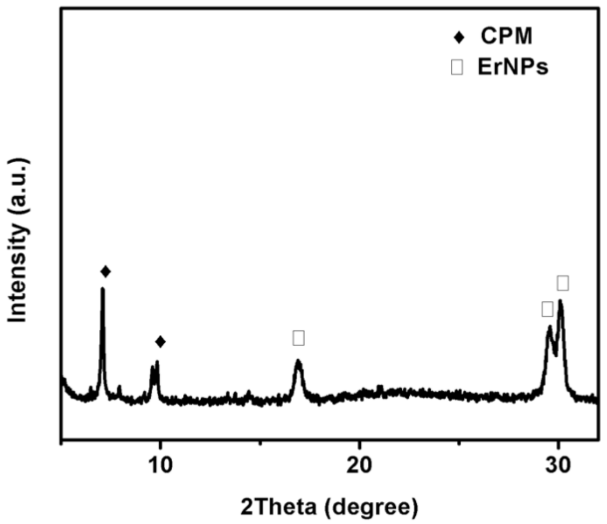

Figure S5. XRD patterns of ECPM nanohybrids.

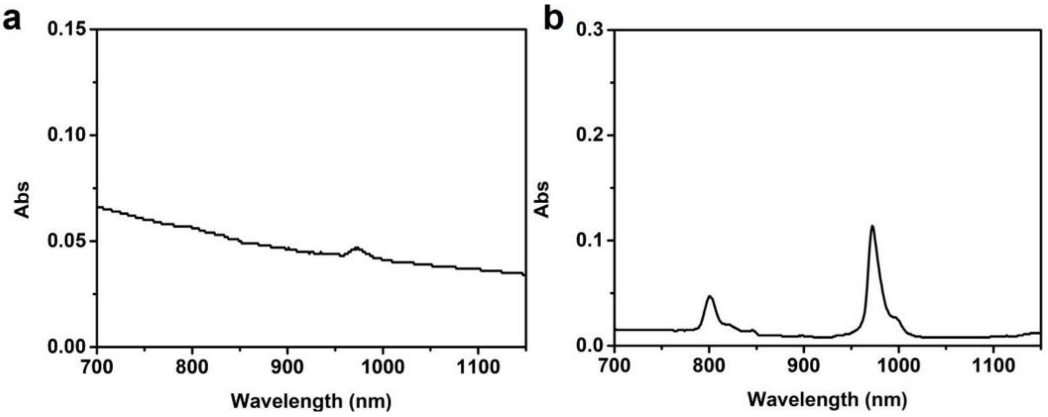

Figure S6. Absorption spectra of (a) ErNPs solution and (b) $ErCl_3$ solution.

As showed in Figure S6, the ErNPs displayed a typical absorption peak within 960-990 nm, which was stronger than that around 808 nm. Therefore, the ErNPs demonstrated stronger UCL and DLC emission intensities under 980 nm laser excitation than that excited by 808 nm laser.

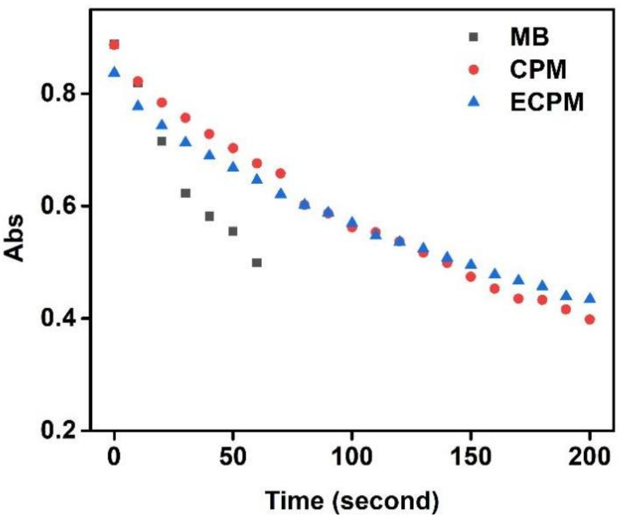

Figure S7. The singlet oxygen ($^1O_2$) quantum yields of CPM and the ECPM hybrid system.

The quantum yields were calculated by the above equation. Based on the above formula, the quantum yield is calculated as φ_{Δ}(CPM) = 0.423, φ_{Δ}(ECPM) = 0.404.

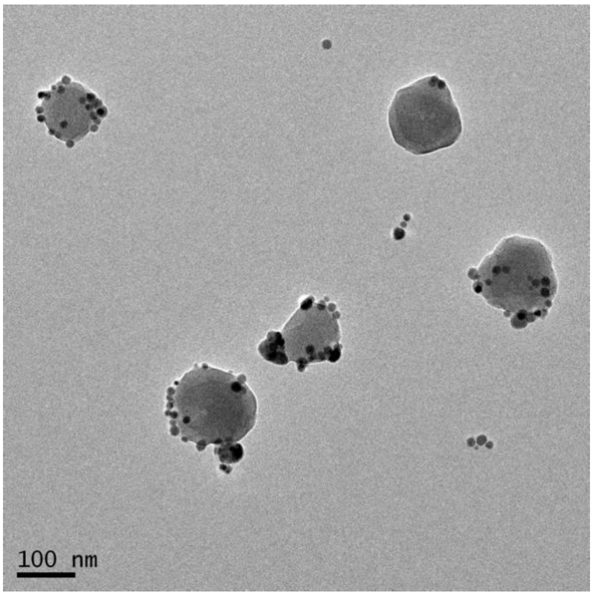

Figure S8. TEM image of ECPM after DNA conjugation.

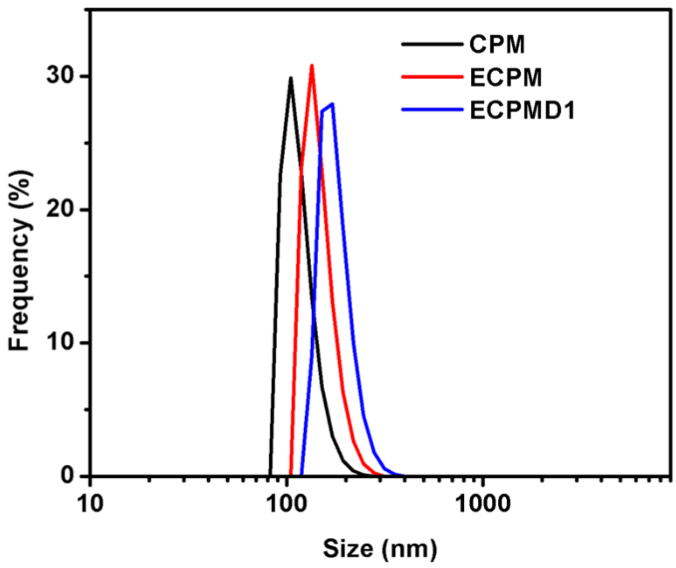

Figure S9. The hydrodynamic sizes of the CPM, ECPM before and after DNA modification.

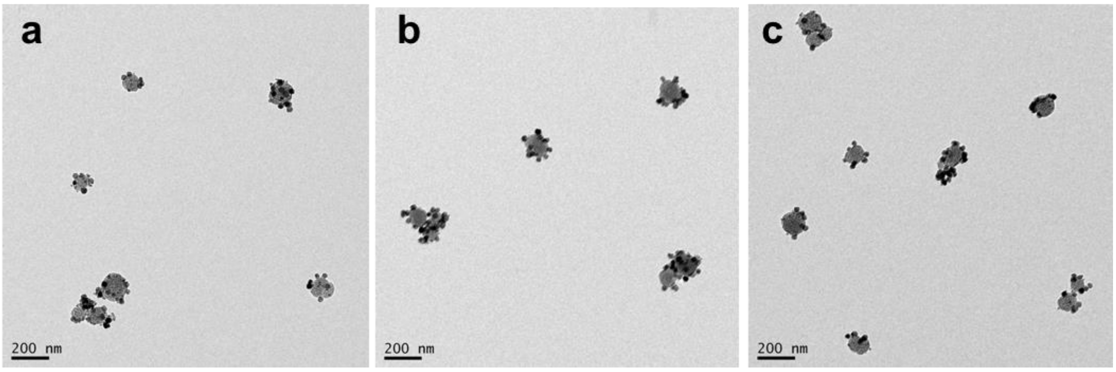

Figure S10. (a) ECPMD1 in pH 7.4 buffer solution. (b) ECPMD2 in pH 7.4 and (c) pH 6.5 buffer solution.

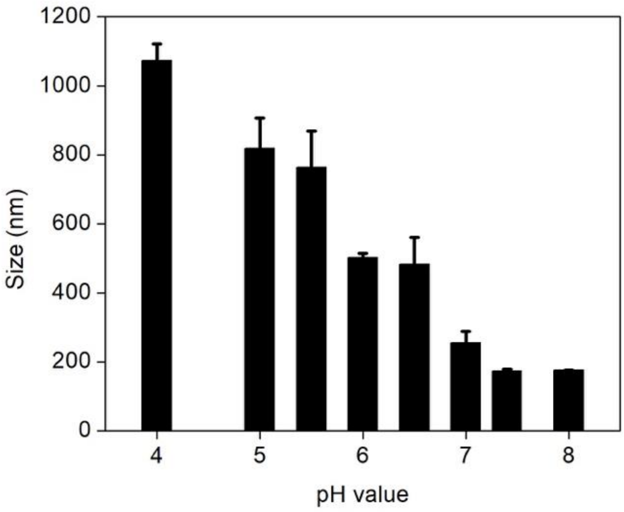

Figure S11. The hydrodynamic sizes of the ECPMD1 at different pH solution. Data are presented as means ± SD (n = 3).

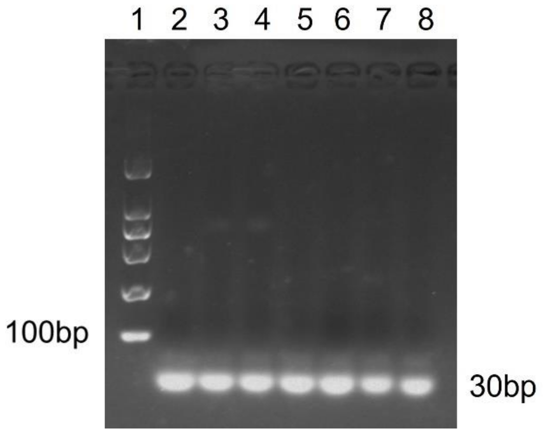

Figure S12. Agarose gel (2%) analysis of i-motif DNA at different condition (lane 1: DNA ladder; lane 2: PBS (pH 7.4); lane 3: 10% FBS (pH 7.4); lane 4: 20% FBS (pH7.4); lane 5: PBS (pH 6.8); lane 6: 10% FBS (pH 6.8); lane 7: 20% FBS (pH6.8); lane 8: i-motif DNA solution). The loading content in each lane is around 5.4 μg.

The stabilizing effects of i-DNA has been confirmed by many literatures. For example, it was reported that the quality of DNA, which was sorted in whole blood, did not change even for 15 days (*PLoS One*, 2017, 12, e0184692). In addition, numerous reports indicated that the DNA-based nanostructures demonstrate enhanced the stability of DNA linkers in biological solutions, which have great promise in biomedical applciations (*Science* 2006, 312, 1027; *J. Am. Chem. Soc.*, 2014, 136, 7261-7264; *J. Am. Chem. Soc.*, 2019, 141, 2215-2219; *Anal. Chem.*, 2019, 91, 3604-3610; *Adv. Sci.*, 2021, 8, 2003775). In this work, we tested the stability of i-DNA in FBS by agarose gel analysis to mimic the *in vivo* environment (Figure R10). In addition, the i-motif DNA we used have cytosine-richened sequences (TTAACAAATTATATTATTCCCCTTTTCCCC, the C/G ratio is 30%), high content of guanine/cytosine bonds is considered to have a good stability.

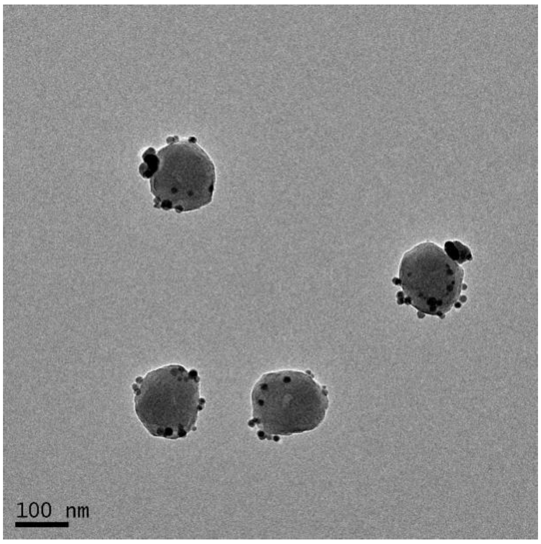

Figure S13. TEM image of ECPMD1 in solution for 14 days.

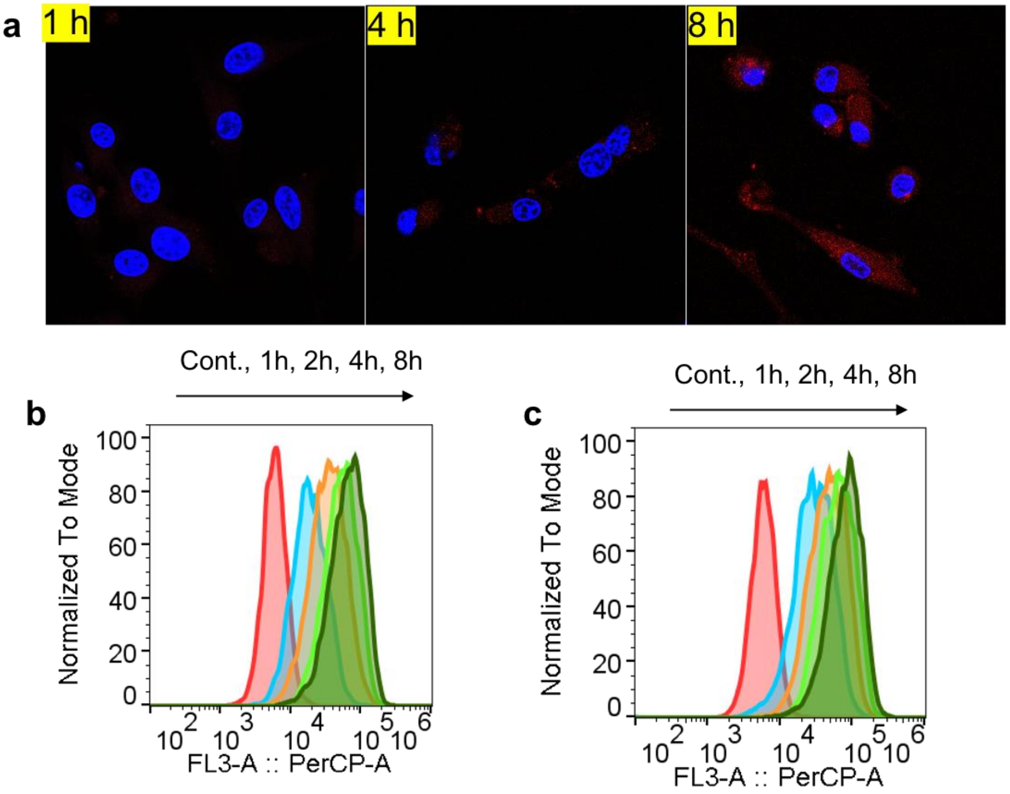

Figure S14. (a) Confocal fluorescence images of the ECPMD1 cultured cells at different time points. The red fluorescence signal is from the CPM. Flow cytometric analysis of cellular uptake of (b) ECPMD2 and (c) ECPMD1 in U87MG cells at different time points.

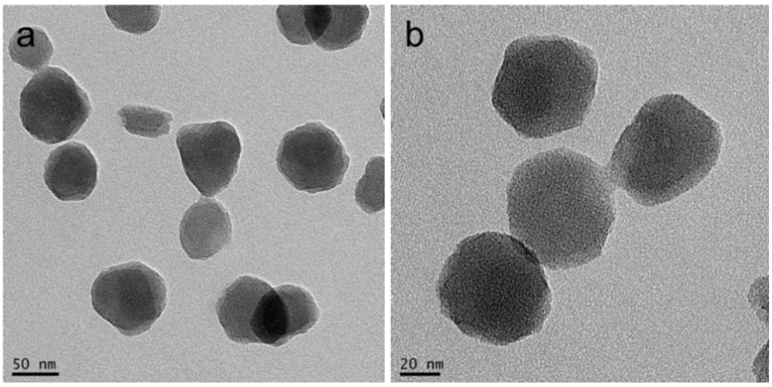

Figure S15. TEM images of TPM at (a) low and (b) high magnifications.

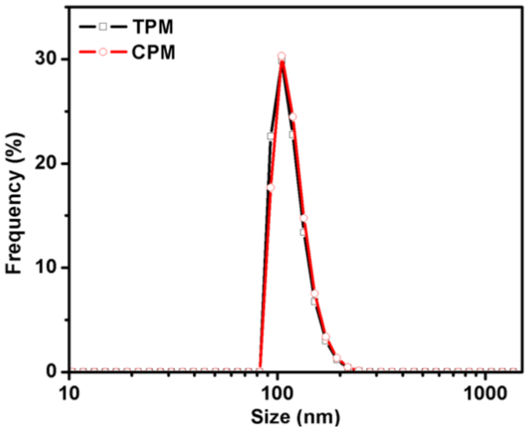

Figure S16. The hydrodynamic diameters of CPM and TPM. The results showed that the as-synthesized CPM and TPM had similar sizes.

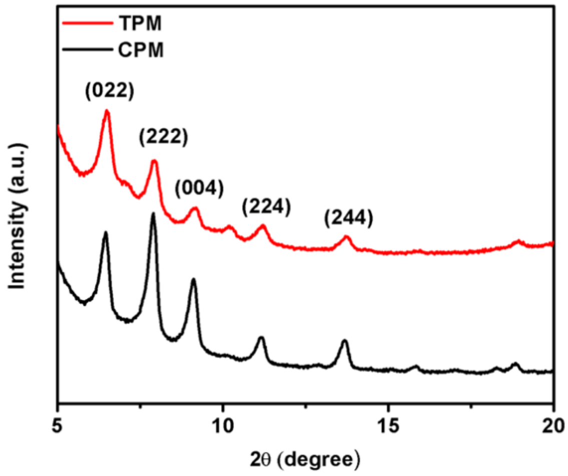

Figure S17. Powder X-ray diffraction pattern of CPM and TPM. The results showed that the XRD patterns of both CPM and TPM matched well with that of PCN-224.

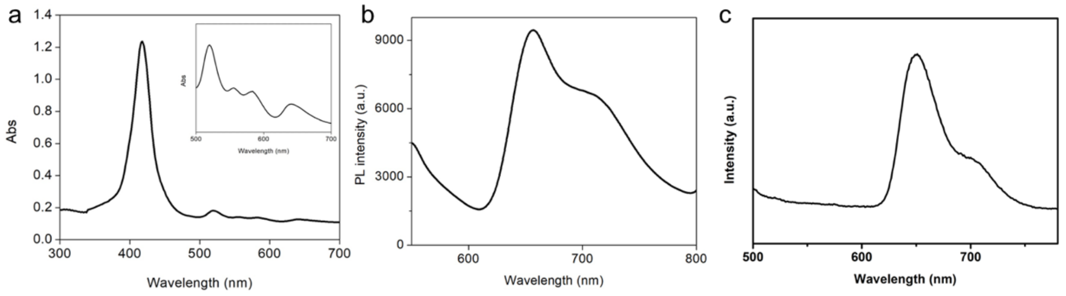

Figure S18. (a) UV-vis spectra and (b) PL spectra (Ex: 420 nm) of TPM. (c) PL spectra (Ex: 420 nm) of CPM.

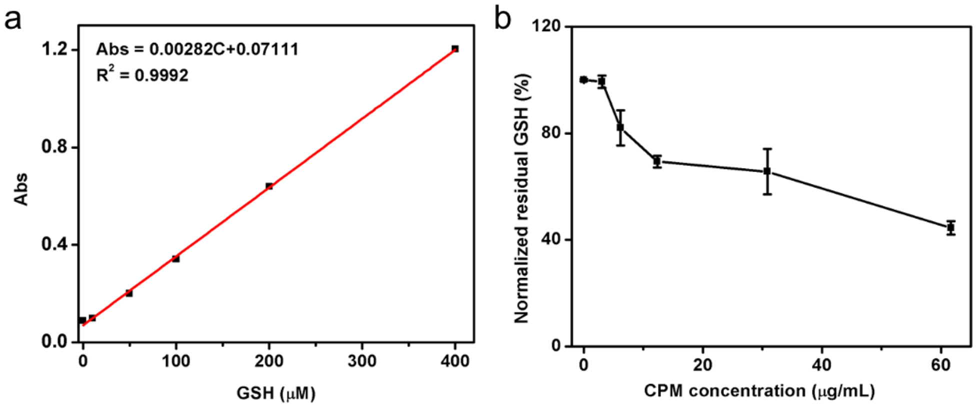

Figure S19. (a) UV-vis absorption standard curve of GSH using DNTB as a sensor. (b) The residual GSH concentration in the GSH/CPM mixture solution detected by DNTB.

Detection of GSH depletion detected by Ellman's reagents: The depletion of GSH was detected by Ellman's reagents. In detail, 5 μL of 5,5′-dithiobis(2-nitrobenzoic acid) (DTNB) (DMSO, 100 mM) was added into 995 μL of GSH solution (with final concentrations of 0, 3.06, 6.17, 12.33, 30.83, and 61.65 μg/mL). After being mixed for 2 min, the absorbance of the solutions at 405 nm was measured using a microplate reader. According to the absorption value, the standard curve of GSH could be obtained. 100 uL of different concentrations of nanoparticle solutions were mixed with 10 μL of 100 mM GSH for 10 min, and then 2 μL of DTNB (100 mM, DMSO) was added to the above 110 μL mixture solution. After standing for 2 min, the absorbance (A_x) of the mixture was measured. The following formula was used to calculate the consumption of GSH (C): $C = (A_x - A_{MOFs})/(A_{GSH} - A_{MOFs}) \times C_0$.

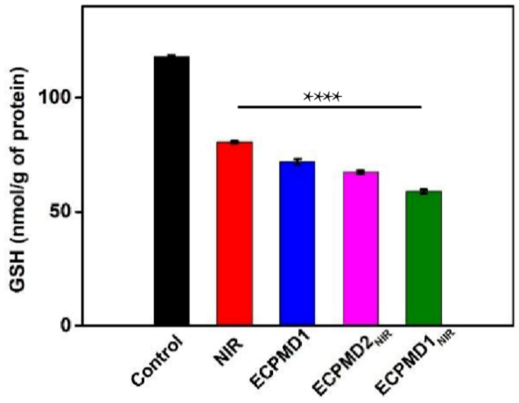

Figure S20. The GSH concentration in U87MG cells with different treatments Data are presented as means ± SD, n = 3. *p < 0.05, **p < 0.01, ***p < 0.001, ****p < 0.0001, by one-way ANOVA with Tukey's post hoc test were considered.

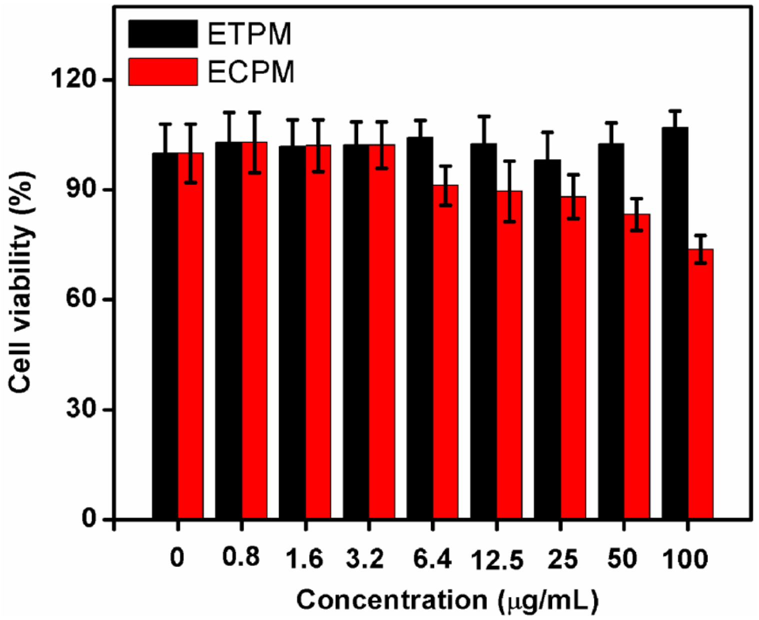

Figure S21. Viability of the U87MG cells treated with different concentrations of ETPM and ECPM. Data are presented as means ± SD, n = 5).

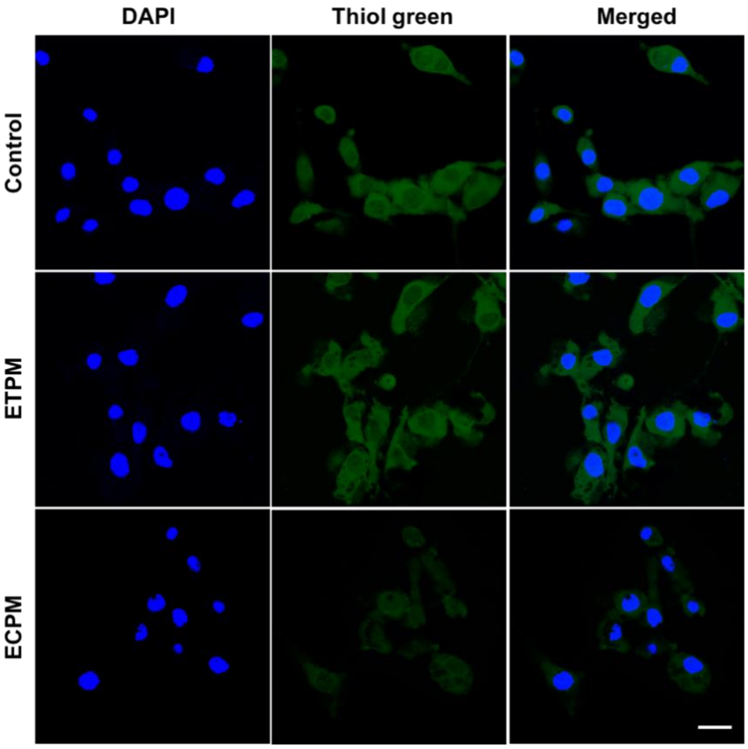

Figure S22. The intracellular GSH level of the cells after different treatments. Scale bar: 20 μM.

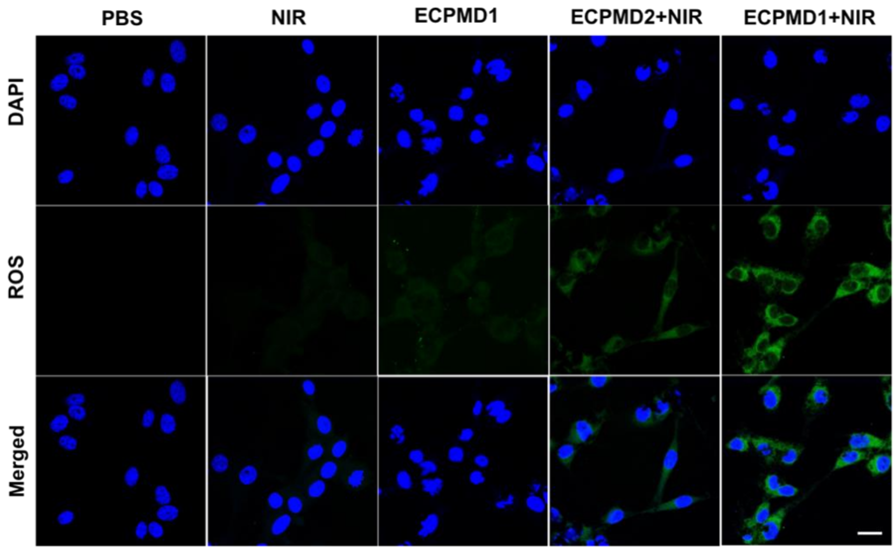

Figure S23. The intracellular ROS level of the cells after different treatments. Scale bar: 20 μM.

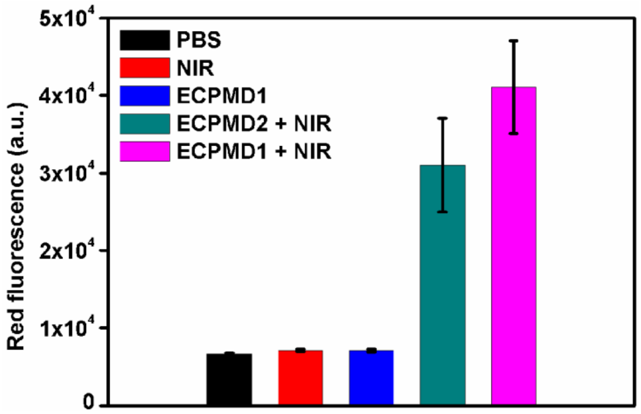

Figure S24. Quantitative analysis of cell death rate by red fluorescence. Data are presented as means ± SD, n = 3.

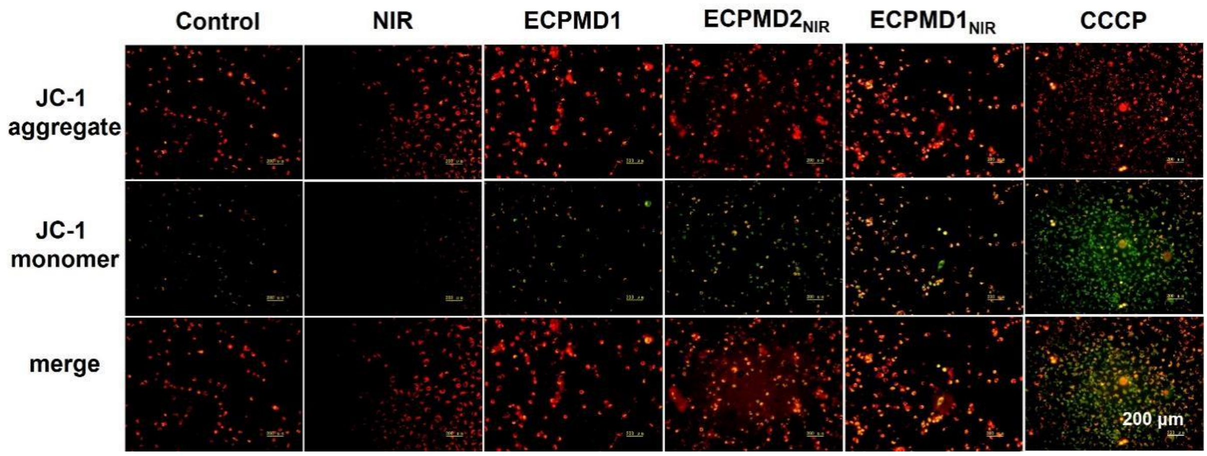

Figure S25. The JC-1 staining of U87MG cells with different treatments (CCCP: Mitochondrial oxidative phosphorylation uncoupler).

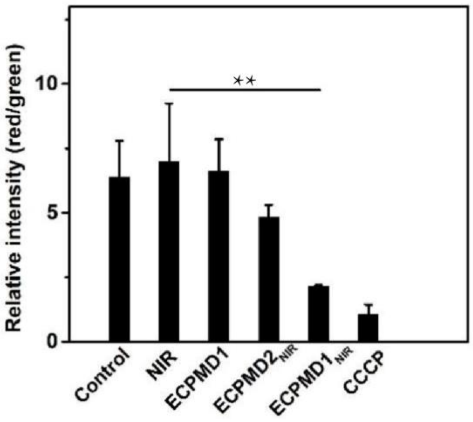

Figure S26. The graphical representation of the ratio of JC-1 aggregates to JC-1 monomers (ratio of 590:530 nm emission intensity). Data are presented as means ± SD, n = 3. $^{*}p < 0.05$, $^{**}p < 0.01$, $^{***}p < 0.001$, $^{****}p < 0.0001$, by one-way ANOVA with Tukey's post hoc test were considered.

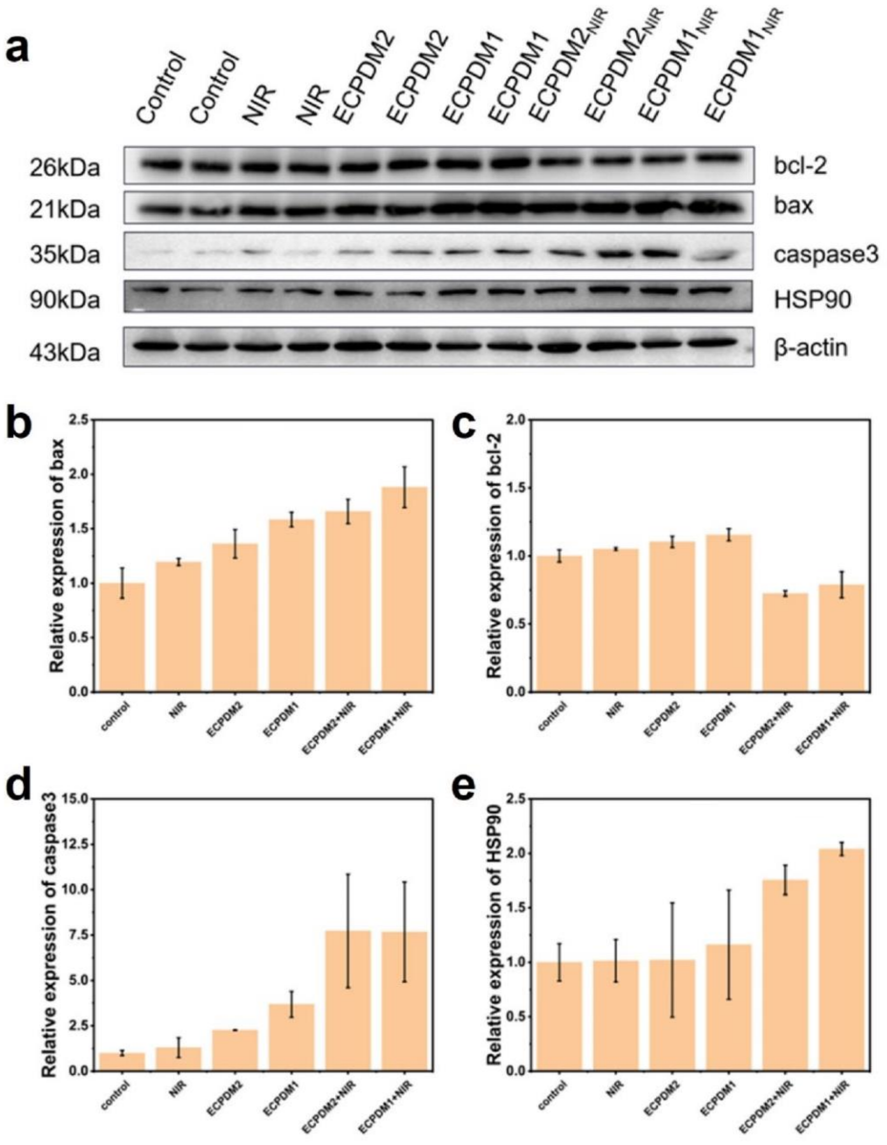

Figure S27. Western blot (WB) analysis of proteins obtained from U87MG cells with different treatments (mean ± SD., n = 2): (a) differential expression patterns of proteins *via* WB analysis of U87MG cell lysate; (b) relative expression of bcl-2; (c) relative expression of bax. (d) relative expression of caspase3; (e) relative expression of HSP90.

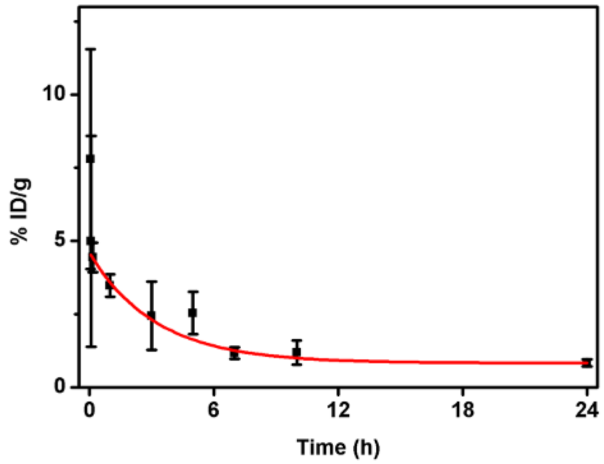

Figure S28. Blood circulation profile of ECPMD1 NPs in mice after i.v. injection, which was determined by ICP-measured Zr^{4+} concentration in blood samples. Data are presented as means ± SD, n = 3.

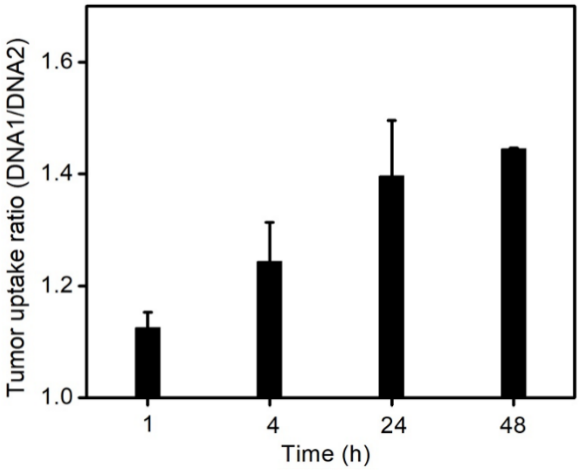

Figure S29. Tumor uptake ratio of ECPMD1 to ECPMD2 over time (based on the PET results). Data are presented as means ± SD, n = 3.

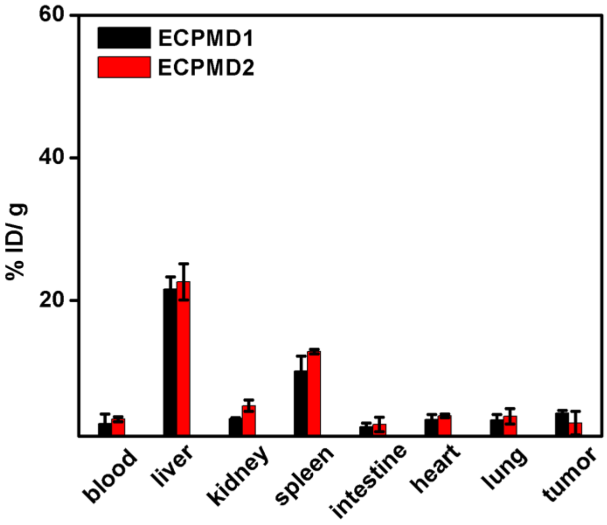

Figure S30. Biodistribution of ^{64}Cu-labeled ECPMD1 and ECPMD2 by PET scan. The mice were injected with the ^{64}Cu-doped NPs, and the primary tissues were collected at 48 h p.i. for region of interest analysis. Ex vivo biodistribution quantification was carried out at 48 h p.i. using a gamma counter. Data are presented as means ± SD, n = 3.

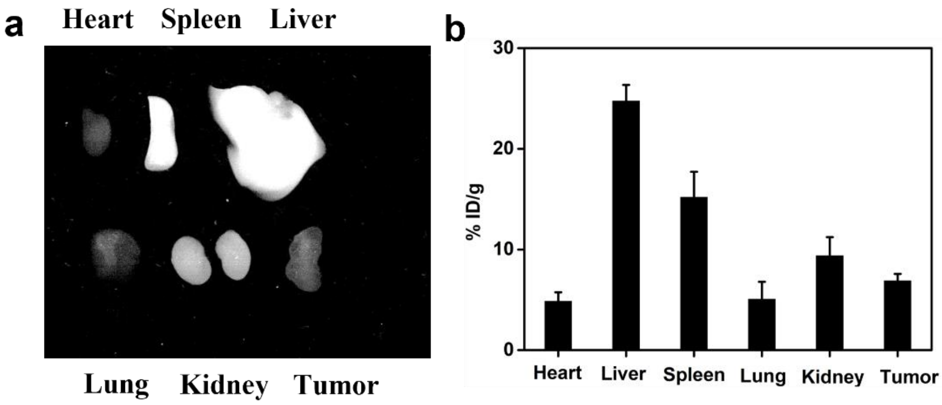

Figure S31. (a) Photo images of major organ observed by NIR-II imaging. The mice were injected with ECPMD1, and the primary tissues were collected at 48 h p.i. (b) *Ex vivo* biodistribution quantification was carried out at 48 h p.i. using ICP-MS, which demonstrated similar results with that in Figure S30. Data are presented as means ± SD, n = 3.

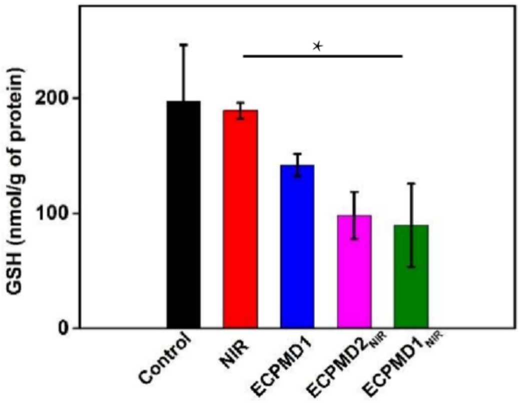

Figure S32. The GSH concentration in tumor tissues after different treatments. Data are presented as means ± SD, n = 3. $^{*}p < 0.05$, $^{**}p < 0.01$, $^{***}p < 0.001$, $^{****}p < 0.0001$, by one-way ANOVA with Tukey's post hoc test were considered.

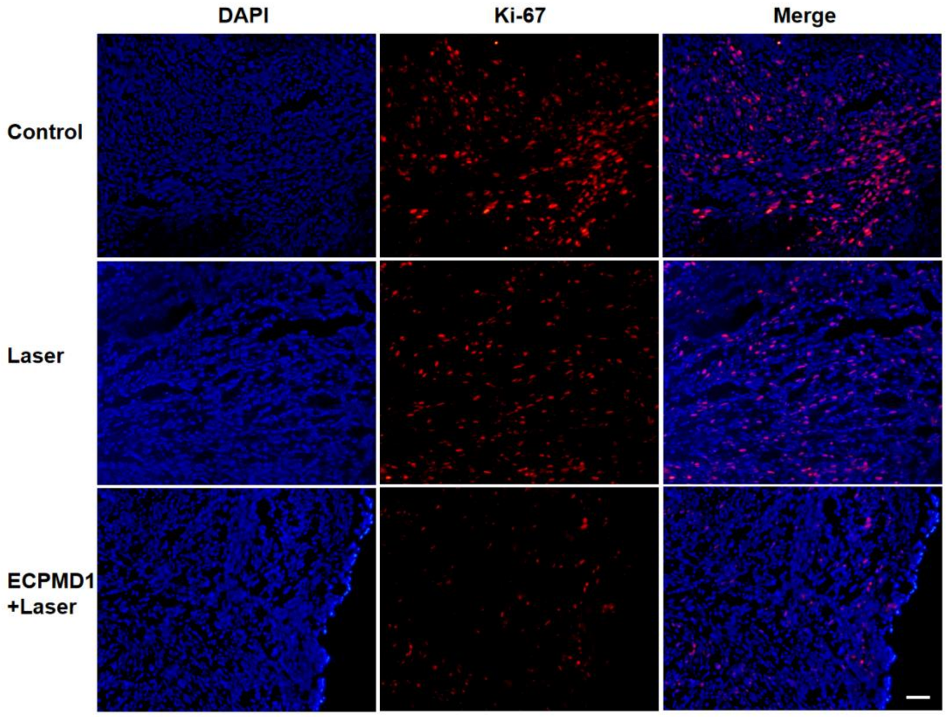

Figure S33. Ki67 staining images of the tumor sections after various treatments at 24 h p.i., scale bar: 50 μm.

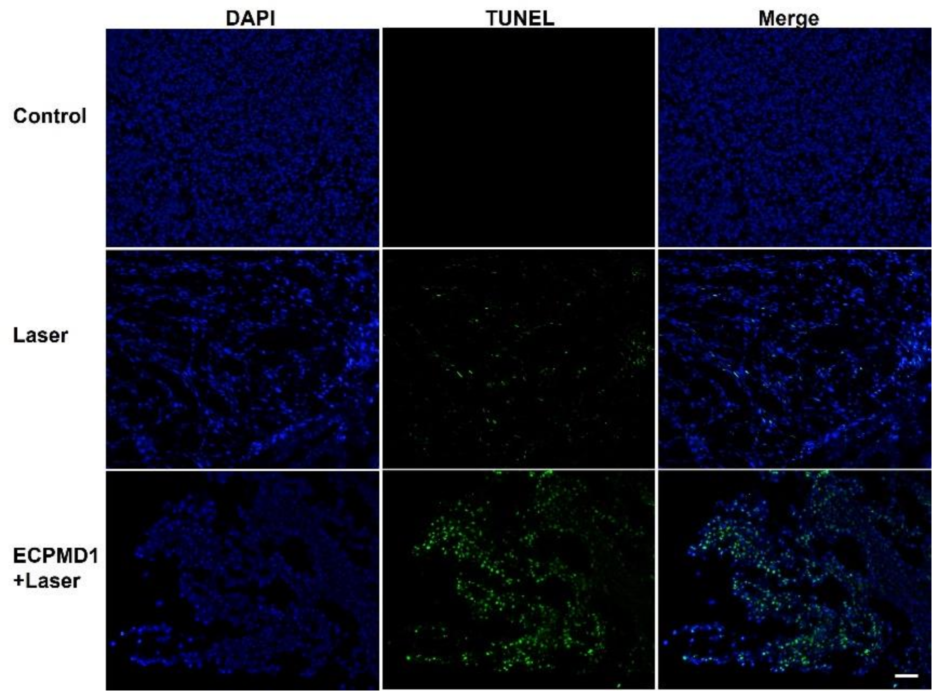

Figure S34. TUNEL staining images of tumor sections after various treatments at 24 h p.i., scale bar: 50 µm.

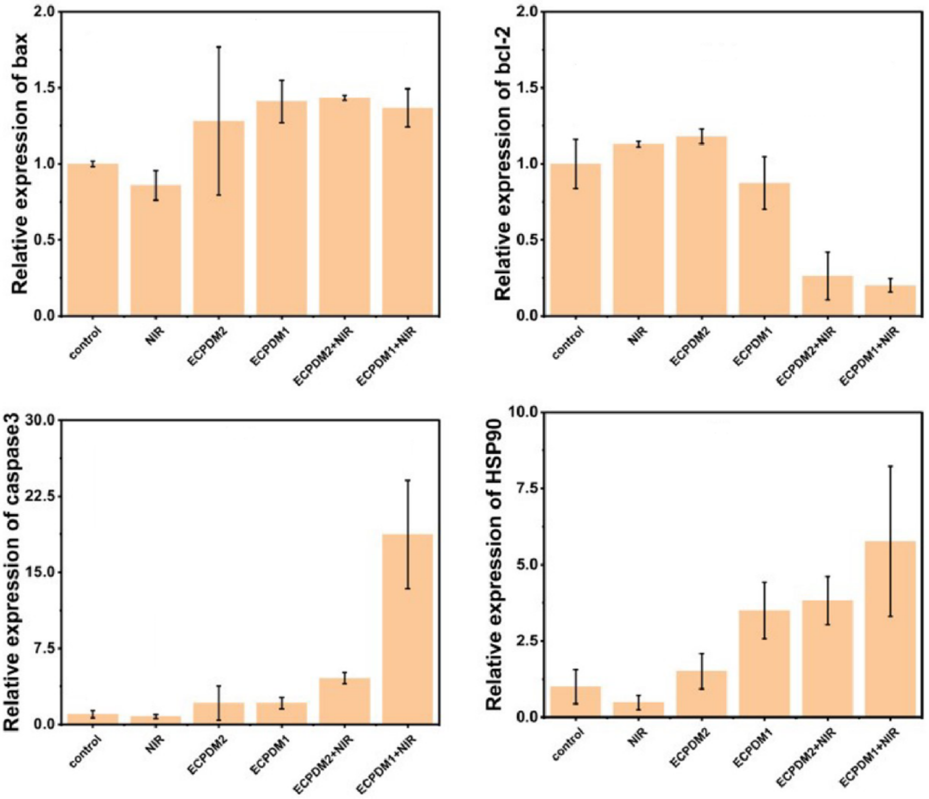

Figure S35. Relative expression of bcl-2/bax/caspase3 and HSP90 in U87MG tumor of different treatment groups (mean ± SD, n = 2).

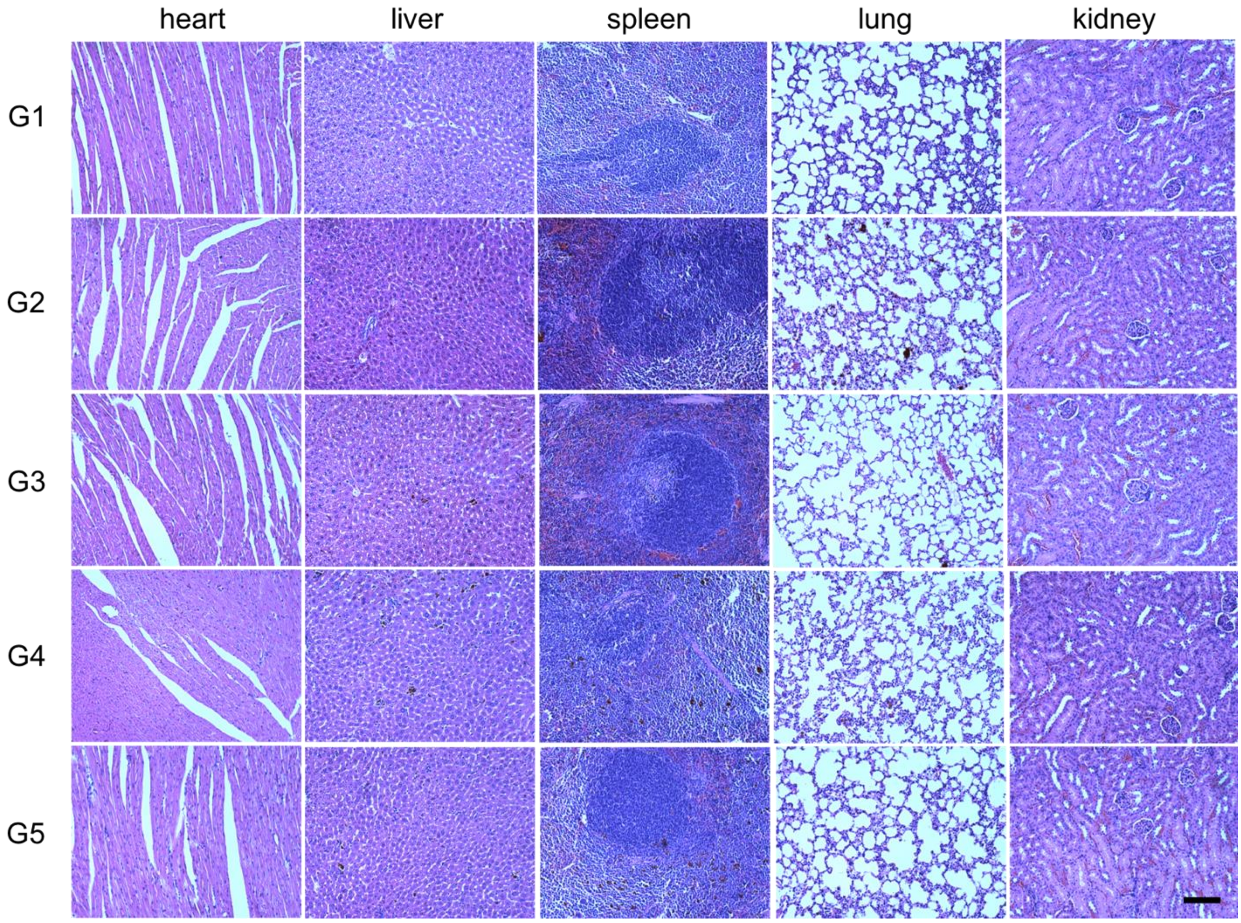

Figure S36. H&E staining on major organ tissues of mice after the PDT treatment. Scale bar, 100 µm.

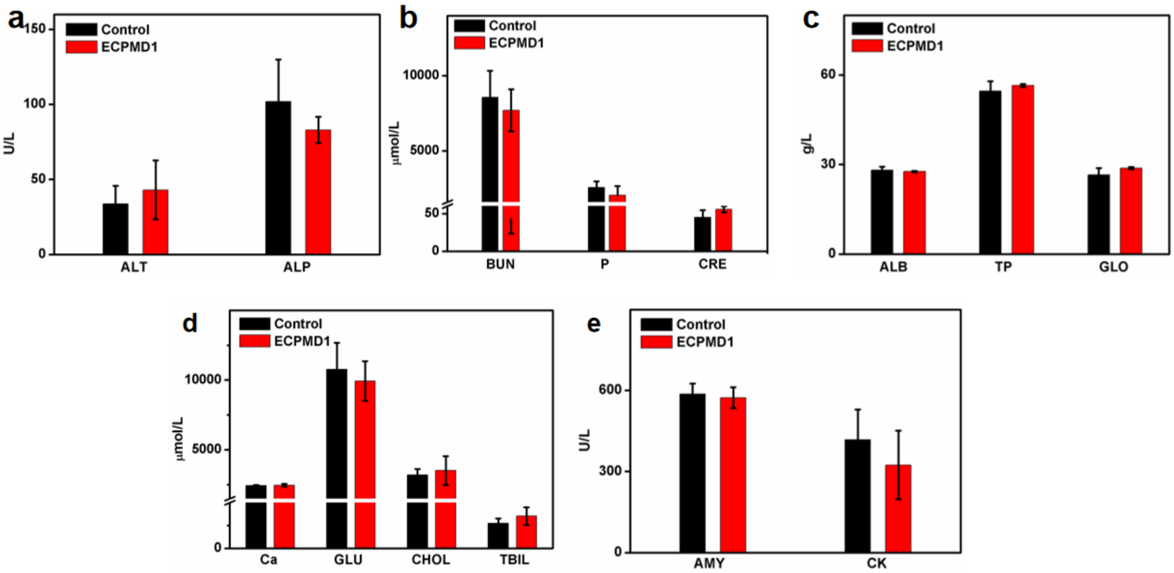

Figure S37. Blood biochemical analysis of (a) ALT, ALP, (b)BUN, P, CRE, (C) ALB, TP, GLO, (e) Ca, GLU, CHOL, TBIL, (f)AMY and CK. Data are presented as means ± SD, n = 3.

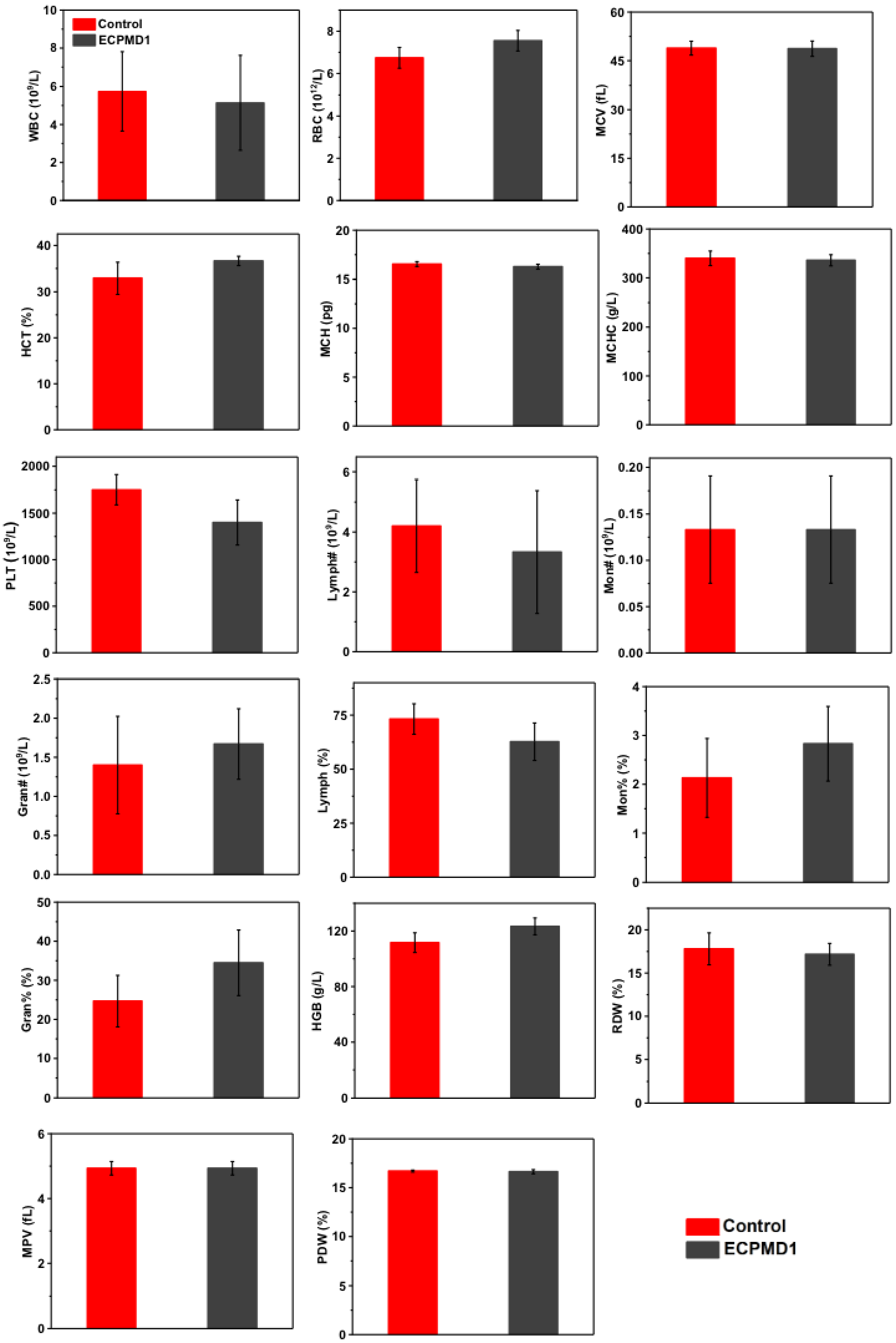

Figure S38. Blood routine assay test results. Data are presented as means ± SD, n = 3.

References

[1] R. Tian, Q. Zeng, S. Zhu, J. Lau, S. Chandra, R. Ertsey, K. S. Hettie, T. Teraphongphom, Z. Hu, G. Niu, D. O. Kiesewetter, H. Sun, X. Zhang, A. L. Antaris, B. R. Brooks, X. Chen. *Sci. Adv.*, **2019**, *5*, eaaw0672;

[2] R. Tian, H. Ma, Q. Yang, H. Wan, S. Zhu, S. Chandra, H. Sun, D. O. Kiesewetter, G. Niu, Y. Liang, X. Chen. *Chem. Sci.*, **2019**, *10*, 326-332;

[3] L. Li, Z. Yang, S. Zhu, L. He, W. Fan, W. Tang, J. Zou, Z. Shen, M. Zhang, L. Tang, Y. Dai, G. Niu, S. Hu, X. Chen. *Adv. Mater.*, **2019**, *31*, 1901187.

[4] Johnson, N. J. J., He, S., Diao, S., Chan, E. M., Dai, H., Almutairi, A. *J. Am. Chem. Soc.*, 2017, 139, 3275-3282.

[5] Wang, S., McGuirk, C. M., Ross, M. B., Wang, S., Chen, P., Xing, H., Liu, Y., Mirkin, C. *J. Am. Chem. Soc.*, 2017, 139, 9827-9830.

[6] Z. Zhou, H. Wu, R. Yang, A. Xu, Q. Zhang, J. Dong, C. Qian, M. Sun. *Sci. Adv.*, 2020, 6, eabc4373.
